# Endocrine disrupting chemicals entering European rivers: Occurrence and adverse mixture effects in treated wastewater

**DOI:** 10.1016/j.envint.2022.107608

**Published:** 2022-12

**Authors:** Saskia Finckh, Sebastian Buchinger, Beate I. Escher, Henner Hollert, Maria König, Martin Krauss, Warich Leekitratanapisan, Sabrina Schiwy, Rita Schlichting, Aliaksandra Shuliakevich, Werner Brack

**Affiliations:** a Department of Effect-Directed Analysis, UFZ – Helmholtz Centre for Environmental Research, Leipzig, Germany; b Department of Evolutionary Ecology and Environmental Toxicology, Goethe University, Frankfurt am Main, Germany; c Department of Biochemistry and Ecotoxicology, Federal Institute for Hydrology – BfG, Koblenz, Germany; d Department of Cell Toxicology, UFZ – Helmholtz Centre for Environmental Research, Leipzig, Germany; e Environmental Toxicology, Department of Geosciences, Eberhard Karls University, Tübingen, Germany; f Environmental Toxicology Unit – GhEnToxLab, Faculty of Bioscience Engineering, Ghent University, Ghent, Belgium

**Keywords:** Endocrine disrupting chemicals (EDCs), Wastewater treatment plant (WWTP) effluents, Chemical target analysis, Effect-based analysis, Water quality assessment, Effect based trigger values (EBTs)

## Abstract

In the present study on endocrine disrupting chemicals (EDCs) in treated wastewater, we used chemical and effect-based tools to analyse 56 wastewater treatment plant (WWTP) effluents from 15 European countries. The main objectives were (i) to compare three different receptor-based estrogenicity assays (ERα-GeneBLAzer, p-YES, ERα-CALUX®), and (ii) to investigate a combined approach of chemical target analysis and receptor-based testing for estrogenicity, glucocorticogenic activity, androgenicity and progestagenic activity (ERα-, GR-, AR- and PR-GeneBLAzer assays, respectively) in treated wastewater. A total of 56 steroids and phenols were detected at concentrations ranging from 25 pg/L (estriol, E3) up to 2.4 μg/L (cortisone). WWTP effluents, which passed an advanced treatment via ozonation or via activated carbon, were found to be less contaminated, in terms of lower or no detection of steroids and phenols, as well as hormone receptor-mediated effects. This result was confirmed by the effect screening, including the three ERα-bioassays. In the GeneBLAzer assays, ERα-activity was detected in 82 %, and GR-activity in 73 % of the samples, while AR- and PR-activity were only measured in 14 % and 21 % of the samples, respectively. 17β-estradiol was confirmed as the estrogen dominating the observed estrogenic mixture effect and triamcinolone acetonide was the dominant driver of glucocorticogenic activity. The comparison of bioanalytical equivalent concentrations (BEQ) predicted from the detected concentrations and the relative effect potency (BEQ_chem_) with measured BEQ (BEQ_bio_) demonstrated good correlations of chemical target analysis and receptor-based testing results with deviations mostly within a factor of 10. Bioassay-specific effect-based trigger values (EBTs) from the literature, but also newly calculated EBTs based on previously proposed derivation options, were applied and allowed a preliminary assessment of the water quality of the tested WWTP effluent samples. Overall, this study demonstrates the high potential of linking chemical with effect-based analysis in water quality assessment with regard to EDC contamination.

## Graphical abstract

**Figure:**
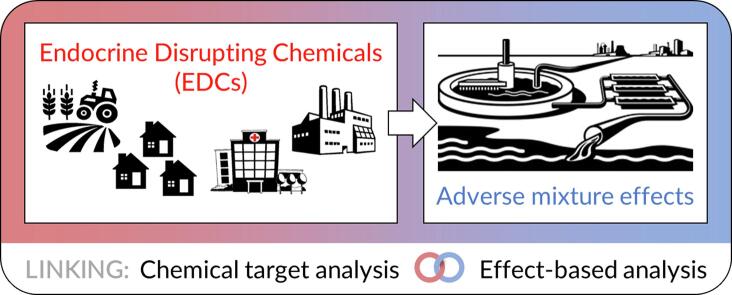

## 1 Introduction

Increasing numbers of chemicals of emerging concern (CECs) are detected in wastewater treatment plant effluents using analytical screening methods (Alygizakis et al., 2019, Gago-Ferrero et al., 2020). In a Europe-wide study on 56 European WWTP effluents we identified 366 CECs, assessed mixture risks and prioritized components based on acute toxicity to aquatic organisms (Finckh et al., 2022). This assessment indicated substantial toxic risks for crustaceans and algae for most of the WWTPs while low risks to fish were estimated. This finding appears to be in contrast to the significant declines of freshwater fish populations that have been observed worldwide (Duncan and Lockwood, 2001, Santos et al., 2017) and to many studies that indicate the impact of chemical pollution on fish using biomarker responses (Santos et al., 2017, Schmitz et al., 2022). Particularly downstream of WWTP discharges, there are clear indications of impaired fish reproduction due to chemical pollution with endocrine disrupting chemicals (Jobling et al., 1998, Schmitz et al., 2022, Streck, 2009, Sumpter and Johnson, 2008, Weitere et al., 2021). Natural and synthetic steroids and some phenolic compounds often dominate endocrine disruption in European surface and wastewaters (Creusot et al., 2014, Hashmi et al., 2018, Hashmi et al., 2020). These highly potent compounds are hardly detectable with state-of-the-art chemical screening methods due to insufficient detection limits but demand for more specific sensitive analytical or bioanalytical tools (Labadie and Budzinski, 2005, Wernersson et al., 2015). At the same time, mixture assessment approaches based on acute toxicity to aquatic organisms typically ignore chemicals that exhibit their impact via endocrine disruption while effect concentrations for endocrine disruption in fish and other aquatic organisms are widely lacking.

Effect-based monitoring using *in vitro* (molecular & cellular level) and *in vivo* (whole organism level) methods has been suggested to fill the gap characterizing the whole mixture via its effects (Brack et al., 2019, Di Paolo et al., 2016), however, without providing information on the compounds causing the effects. Thus, combining effect-based with chemical screening tools is the most promising approach to detect, unravel and prioritize mixtures and compounds driving adverse effects (Altenburger et al., 2019, Escher et al., 2020b). Specific effect-based methods combined with sensitive chemical analysis of steroids and other related compounds are required to meet these goals in wastewater effluents and surface waters for endocrine disruptors. In a recent interinstitutional study surface and wastewater samples were analysed with sensitive LC-MS/MS measurements and five different *in vitro* estrogen receptor (ERα) assays (ERα-CALUX, p-YES, MELN, HeLa-9903, and ERα-GeneBlazer) (Kase et al., 2018, Könemann et al., 2018). Bioassay activities could be explained mainly by the detected concentrations of natural and synthetic estrogens (i.e. 17α-ethinyl estradiol (EE2), 17β-estradiol (E2), and estrone (E1)). Another interinstitutional study compared the intra- and inter-day variability of E2-equivalent concentration (EEQ) measurements using five estrogen bioassays (YES, ERα-CALUX®, MELN, T47D-KBluc and ERα-GeneBLAzer) with regard to their applicability as effect-based tools in environmental monitoring (Kunz et al., 2017). The average coefficient of variation of EEQ concentrations for the five assays and all samples was 32 %. Currently, ERα mediated effects are the best-studied endpoints, followed by effects via androgen receptor (AR) binding (Evans, 2018, Hou et al., 2018). However, also glucocorticoid receptor (GR) and progestogen receptor (PR) mediated activities are getting into the focus of research and monitoring of endocrine disrupting chemicals (Hamilton et al., 2022, Hashmi et al., 2020, Jia et al., 2016, König et al., 2017). Nevertheless, a systematic analysis, assessment and prioritization of larger numbers of steroids and phenolic endocrine disruptors together with effect-based monitoring of whole mixtures in a larger set of European WWTP effluents is still missing and provided in this study.

The present study is a follow-up project on a recent publication by Finckh et al. (2022), in which we combined chemical and effect-based analysis to investigate WWTP effluents from 15 European countries for the presence (concentrations) and potential effects (receptor activities) of endocrine disrupting chemicals (EDCs). To this end, the extensive set of 56 European effluent samples was analysed for 79 natural and synthetic steroids and some selected phenols with known or expected ER-, AR-, PR- and GR-activity. A combined LC-MS/MS and LC-HRMS target analysis approach was used together with nuclear receptor-based *in vitro* assays. For estrogenicity testing three different receptor-based assays were compared. Iceberg modelling (Neale et al., 2015) was used to link receptor-based effects in the ERα-GeneBLAzer assay with chemical-analysis-based endocrine disruption potential. By comparing measured bioanalytical equivalent concentrations (BEQ_bio_) with predicted bioanalytical equivalent concentrations (BEQ_chem_) from detected concentrations and the relative effect potencies (REP) major contributors to WWTP effluent endocrine disruption potential were identified. Effect-based trigger values (EBTs) were developed and discussed to differentiate between acceptable and non-acceptable water quality providing a bioassay-specific threshold (Escher et al., 2018a). Finally, the set of European WWTP effluents was ranked according to their endocrine disruptive potential and evaluated for the impact of advanced wastewater treatment technologies.

## 2 Material and methods

### 2.1 Sampling, sample processing and storage

The sample set comprises a number of 56 effluent extracts from 52 wastewater treatment plants located in 15 European countries, selected for their composition and accessibility (SI, Table B1). Different capacities and conventional treatment technologies are covered, as well as advanced treatment technologies via ozonation (EU032, EU128, and EU130) and activated carbon (EU019). The samples were taken by on-site large volume solid phase extraction (LVSPE), re-dissolved in LC-MS grade MeOH at a relative enrichment factor (REF) of 1000 (i.e. 50 ml) and stored at −20 °C until further analysis (Schulze et al., 2017). In addition to the effluent samples, eleven field blanks and one machine blank were prepared (“processing blanks”, EU201-EU212) for quality control (QC). Field blanks based on filtered water from a pristine stream (Wormsgraben) in the upper Harz Mountains (Germany) were processed according to the same procedure as the effluent samples until the elution process without any sample enrichment step. The machine blank was processed in the laboratory using 5 L of LC-MS grade water (Sigma-Aldrich) containing analytical grade sodium chloride (0.2 g/L, Merck). More details on the investigated set of samples are provided in Finckh et al. (2022).

The effluent extracts were subjected to a clean-up via an aminopropyl column based on Labadie and Budzinski (2005). An aliquot of the processed extracts was derivatized with dansyl chloride to enhance the ionizability of specific phenolic compounds and steroids (Backe, 2015). Further details on the sample processing are provided in the supporting information (Section A1.1).

### 2.2 Chemical target analysis

The samples (effluent extracts in MeOH) were analysed for 79 steroids and phenols by liquid chromatography mass spectrometry (LC-MS). Ketosteroids and phenols including all bisphenols were analysed by LC-MS/MS (QTrap 6500, Sciex) in positive and negative ion mode (ESI+ and ESI−), respectively. Estrogens and some of the phenols were analysed by LC-HRMS (Q-Exactive Plus, Thermo) after derivatisation in ESI+ mode. In all methods internal standards were used to improve the accuracy and precision of quantification. An internal standard (IS) mixture of 39 isotope-labelled compounds (SI, Table B3) was added for quality assurance (QA) prior to the clean-up. Sample extracts concentrated to a REF of 1000 were measured along with calibration standards, which were processed by the same clean-up method (method-matched calibration) and corresponded to a range of 0.1-1000 ng/mL for the phenols and 0.01-100 ng/mL for the steroids (SI, Table A1).

The output data were further processed using the vendor software *MultiQuant* 3.0.3 (Sciex) and *TraceFinder* 5.1 (Thermo). Final concentrations refer to the extracts (i.e., LVSPE recovery was not considered). This allowed the comparison with the measured effects, which were performed in the extracts and not in the original water samples. The recoveries of the LVSPE method of all analysed compounds, which allow for a back-calculation of concentrations in the water sample are listed in the supporting information (Table B5); the average recovery is 0.89. The supporting information provides further details on the chemical target analysis including a summary of QA/QC measures (SI, Section A1.2, Tables B2–B4).

### 2.3 Effect-based analysis

Three different ERα-assays were applied for estrogenicity testing, including the planar Yeast Estrogen Screen (p-YES) test and the Chemical Activated Luciferase Gene Expression (ERα-CALUX®) assay, both without the previously described aminopropyl clean-up step of the samples (effluent extracts in MeOH). The third ERα assay was the GeneBLAzer™ ERα-UAS-bla GripTite™ assay, performed twice: Once prior to and once after an additional clean-up step. The pre-treated samples (incl. clean-up step) were additionally investigated for activation of the glucocorticoid- (GR), androgen- (AR) and progestogen receptors (PR) in the respective GeneBLAzer reporter gene assay (GR-UAS-bla HEK 293 T assay, AR-UAS-bla GripTite™ and PR-UAS-bla HEK 293 T assay). All assays were performed in agonistic mode only. More detailed information on the effect-based tools used including a summary of QA/QC measures can be found in the supporting information (Section A1.3).

#### 2.3.1 p-YES bioassay

The Yeast Estrogen Screen (YES) bioassay based on yeast cells according to McDonnell et al. (1991) was performed on silica-surface plates (p-YES) (Buchinger et al., 2013) for high performance thin-layer chromatography (HPTLC) after chromatographic separation of the sample. The samples were applied on 10 cm × 20 cm silica gel 60 F_254_ HPTLC plates (Merck, Darmstadt) using the automatic TLC sampler ATS 4 (CAMAG, Muttenz). Estrone (E1), 17β-estradiol (E2), 17α-ethinylestradiol (EE2) and estriol (E3) were sprayed in 5 mm bands as reference compounds in separated lanes at different levels ranging from 1-10 pg for EE2 and E2, 10-100 pg for E1 and 100-1000 pg for E3. Depending on their estrogenic potential, the samples were applied in volumes between 10 and 50 µL. After evaporation of the solvent, the HPTLC-plates were developed with 100 % methanol up to 20 mm for focussing and further developed up to 90 mm with ethylacetate/chloroform/petroleum fraction 20:55:25 in the automated developing chamber AMD 2 (CAMAG, Muttenz). Finally, the yeast cells were applied to the HPTLC-plate by spraying (Schoenborn et al., 2017).

HPTLC signals from fluorescence measurements were recorded in arbitrary units (AU) and signals of the reference compound E2 were used to quantify signals detected in the samples in terms of E2-equivalence (EEQ_p-YES_). EEQ_p-YES_ in ng/L were calculated using the respective application volumes of the samples and the enrichment factor of the sample. Further information on the performed p-YES as well as an example image of an HPTLC plate can be found in the supporting information (Section A1.3.1, Fig. A1).

#### 2.3.2 ERα-CALUX® bioassay

In addition, estrogenic activity was detected using the ERα-CALUX® bioassay with licensed cells (BioDetection Systems B.V., the Netherlands) according to ISO 19040-3:2018(en) (ISO, 2008) and as detailed in Shuliakevich et al. (2022). The ERα-CALUX® cells are human osteoblastic osteosarcoma cells with a transfected human estrogen α receptor (hERα). A ligand-receptor complex moves to the nucleus-internal responsive element, which controls the reporter gene's expression for the luciferase enzyme the activity which is quantified by means of relative light units (RLU) due to bioluminescence after the addition of the luciferase-specific substrate luciferin. The measured RLU can be translated into equivalents of the reference substance E2 (EEQ_CALUX_). The limit of detection (LOD) within each tested plate was calculated as the average of RLU values within the blank of the standard row plus its threefold standard deviation. The limit of quantification (LOQ) was calculated as the threefold LOD.

Prior to the ERα-CALUX® assay, sample dilutions with cytotoxic effects were excluded using the MTT (3-(4,5-dimethyltetrazolium-2-yl)-2,5-diphenyltetrazolium bromide) assay (Mosmann, 1983). Vital ERα-CALUX® cells metabolise water-soluble MTT salt to insoluble formazan. Formazan building rate can be measured photometrically (492 nm).

#### 2.3.3 GeneBLAzer bioassay

GeneBLAzer bioassay measured ERα-, GR-, AR- and PR-activity. The cell lines of all four GeneBLAzer assays are based on human embryonic kidney cells (HEK293), containing the DNA-binding domain of GAL4 gene and stably transfected with a β-lactamase reporter.

The assay was performed according to König et al. (2017) with small modifications detailed in the supporting information (Section A1.3.2). In brief, the samples were evaporated by a gentle nitrogen stream and redissolved in assay medium to reach the highest REF at 400 in the dosing vial. A volume of 30 µL cells were seeded and 10 µL of sample was added. Therefore, the REF400 in the dosing vial corresponds to REF100 in the test. Each concentration is measured in duplicate per plate. The stock of reference chemicals were prepared in MeOH, evaporated by a gentle nitrogen stream and reconstituted in the assay medium. Ten serial dilution steps of a factor 2 were done using the Hamilton Robot Microlab Star. The final REF of the samples and processing blanks ranged from 0.1 to 100. After dosing, the cell viability was analysed by observing the cell confluency using the IncuCyte SE Live Cell Analysis System (Essen Bioscience) directly (t0h) and after 24 h of incubation at 37 °C and 5 % CO_2_. The expression of the hormone pathway related reporter protein β-lactamase was detected by adding fluorescence resonance energy transfer (FRET) substrate, at the excitation wavelength of 409 and 590 nm and emission wavelength of 460 nm (activated response element, blue cell), and 530 nm (inactive response element, green cell). The fluorescence was measured twice, at time zero and after 2 h of incubation in the dark and at room temperature, in order to account for possible interference by autofluorescence of the sample. The ratio of blue to green (B/G) was used to express the effect induced by the samples, which was calculated according to König et al., 2017, Escher et al., 2018b.

In the following chapters on results and discussion, a sample was considered “active” if an EC_10_ could be derived. If no EC_10_ was reported, this was due to the following reasons: (i) the EC_10_ was negative or greater than REF100, (ii) the EC_10_ was masked by cytotoxicity, or (iii) the concentration range did not reach the 10 % effect level (SI, Fig. A4, Tables B8–B11).

### 2.4 Iceberg modelling – BEQ calculation for GeneBLAzer bioassay

The results of the GeneBLAzer bioassays and the chemical target analysis were compared based on the concept of Iceberg Modelling. Here, effect data and chemical concentrations are linked by calculating bioanalytical equivalent concentrations, i.e. BEQ_bio_ and BEQ_chem_, respectively. Details on the concept are provided by Escher et al. (2021).

BEQ_bio_ is the ratio of the EC_10,ref_ and the effect concentration of the whole mixture EC_10,sample_.

(1) $${\mathrm{BEQ}}_{\mathrm{bio}}=\frac{{\mathrm{EC}}_{10,\mathrm{ref}}}{{\mathrm{EC}}_{10,\mathrm{sample}}}$$

The corresponding standard error (SE) is derived based on an error propagation.

(2) $$\mathrm{SE}\left({\mathrm{BEQ}}_{\mathrm{bio}}\right)=\sqrt{{\left(\frac{1}{{\mathrm{EC}}_{10,\mathrm{sample}}}\right)}^{2}\cdot \mathrm{SE}{\left({\mathrm{EC}}_{10,\mathrm{ref}}\right)}^{2}+{\left(\frac{{\mathrm{EC}}_{10,\mathrm{ref}}}{{\mathrm{EC}}_{10,\mathrm{sample}}^{2}}\right)}^{2}\cdot \mathrm{SE}{\left({\mathrm{EC}}_{10,\mathrm{sample}}\right)}^{2}}$$

BEQ_chem_ is the sum of all detected compounds of the measured concentration (c_*i*_) multiplied with the relative effect potency (REP_*i*_) of a compound *i*.

(3) $${\mathrm{BEQ}}_{\mathrm{chem}}=\sum_{i=1}^{n}{\mathrm{BEQ}}_{\mathrm{chem},i}=\sum_{i=1}^{n}{\mathrm{REP}}_{i}\;\cdot c_{i}$$

REP_*i*_ is the fraction of the effect concentration of the reference compound (EC_10,ref_) and the effect concentration of compound *i* (EC_10,*i*_).

(4) $${\mathrm{REP}}_{i}=\frac{{\mathrm{EC}}_{10,\mathrm{ref}}}{{\mathrm{EC}}_{10,i}}$$

The EC_10,ref_ of 17β-estradiol (ERα), dexamethasone (GR), R1881 (AR) and progesterone (PR) were measured along with the samples. The EC_10,*i*_ was derived from single-compound testing. They were available either from previous studies (Escher et al., 2020a, Hashmi et al., 2020) or from new tests conducted as part of the present study. For the compounds for which no effect data were available, EC_10_ values were retrieved from the CompTox database (US-EPA, 2022). An overview of all chemicals included in this study and their EC_10_ and REP_i_ values can be found in the supporting information (Table B6).

BEQ_chem_ and BEQ_bio_ of the ERα-, GR-, AR- and PR-GeneBLAzer assays were expressed as EEQ, DEXA-EQ, R1881-EQ and Progesterone-EQ, respectively. Also, the results of the p-YES and the ERα-CALUX® assays were transformed into BEQ_bio_ and expressed in the unit of EEQ. For a comparison with BEQ_chem_, bioassay-specific REP_i_ values would be required. A direct comparison of BEQ_chem_ and BEQ_bio_ was only performed for the results from the GeneBlazer assays.

### 2.5 Effect-based trigger values for water quality assessment

One can assign a BEQ_chem,*i*_ to each chemical *i* at any concentration (Equation (3)) and this concentration can be the environmental quality standard concentration (EQS_*i*_) defined in the EU Water Framework Directive (European Communities, 2011, Scientific Committee on Health EaER, 2018). The new expression is a compound specific effect-based trigger value (EBT_*i*_), which would be a safe EBT for one chemical *i*.

(5) $${\mathrm{EBT}}_{i}={\mathrm{REP}}_{in\;vitro,i}\cdot {\mathrm{EQS}}_{in\;vivo,i}$$

However, only for very few chemicals EQS_*i*_ are available and hardly any for hormone-active chemicals. As proxies of EQS_*i*_, predicted no-effect concentrations (PNEC_*i*_) or no observed effect concentrations (NOEC_*i*_) for *in vivo* hormone-active effects could be used.

(6) $${\mathrm{EBT}}_{i}={\mathrm{REP}}_{in\;vitro,i}\cdot {\mathrm{PNEC}}_{in\;vivo,i}$$

Transforming Equation (4) and replacing the effect concentration by PNEC*_in__vivo_* leads to Equation (7).

(7) $${\mathrm{PNEC}}_{in\;vivo,i}=\frac{{\mathrm{PNEC}}_{in\;vivo,\mathrm{ref}}}{{\mathrm{REP}}_{in\;vivo,i}}$$

Finally, a new expression for EBT_*i*_ was defined by combining Equation (6) and Equation (7).

(8) $${\mathrm{EBT}}_{i}={\mathrm{PNEC}}_{in\;vivo,\mathrm{ref}}\cdot \frac{{\mathrm{REP}}_{in\;vitro,i}}{{\mathrm{REP}}_{in\;vivo,i}}$$

Equation (8) was used to account for different REPs between *in vivo* and *in vitro*, which forms the basis for the derivation of bioassay-specific effect-based trigger values (EBTs) including all detected and (bioassay-) active compounds.

Escher et al. (2018a) proposed an option, where the mean EBT (Table 1, Core equation) was applied, adjusted by the mean fraction of the individual compounds *i* detected in surface water (Table 1, Option 1). In the present study, we explored if an expansion of the core equation (Equation (9)) and the one weighted by fraction of each chemical detected in the treated wastewater (Equation (10)) could be improved and more chemicals could be included by adding Equation (8) to both formulas. Resulting EBT definitions, Options 2 & 3, are shown in Table 1 (Equations (11) & (12), respectively). The required data for all calculations are given in the supporting information (Tables B6 & B13).

**Table 1:** EBT derivation options, adapted from Escher et al., 2018a, Escher et al., 2018b, Jarosova et al., 2014.

Core equation	Option 1	Option 2	Option 3
Mean EBT of all chemicals at their PNEC or EQS	Exposure-corrected mean EBT (i.e. applying the fraction of each chemical in the mixture f_*i*_ prior to summing up the contribution to the EBT)	“Core equation” + *in vivo/in vitro* correction (i.e. incl. Equation (8))	“Option 1” + *in vivo/in vitro* correction (i.e. incl. Equation (8))
Equation (9)	Equation (10)	Equation (11)	Equation (12)
$\mathrm{EBT}=\frac{\sum_{i=1}^{n}{\mathrm{EBT}}_{i}}{n}$	$\mathrm{EBT}=\sum_{i=1}^{n}f_{i}\cdot {\mathrm{EBT}}_{i}$	$\mathrm{EBT}=\frac{{\mathrm{EBT}}_{\mathrm{ref}}}{n}\overset{n}{\underset{i=1}{\cdot \sum }}\frac{{\mathrm{REP}}_{in\;vitro,i}}{{\mathrm{REP}}_{in\;vivo,i}}$	$\mathrm{EBT}={\mathrm{EBT}}_{\mathrm{ref}}\overset{n}{\underset{i=1}{\cdot \sum }}f_{i}\cdot \frac{{\mathrm{REP}}_{in\;vitro,i}}{{\mathrm{REP}}_{in\;vivo,i}}$
“Option B” in Escher et al., 2018a, Escher et al., 2018b	“Option G” in Escher et al., 2018a, Escher et al., 2018b		
Not used	Proposed previously	New option	New option

### 2.6 Data processing and visualisation

Data analysis and basic bar plots were performed and prepared with Microsoft Excel 2013. Stacked bar plots and Venn diagrams were created in R (version 1.2.1335). All further plots were created in GraphPad Prism (version 9.4.0).

## 3 Results and discussion

### 3.1 Chemical target analysis

**Fig. 1:**
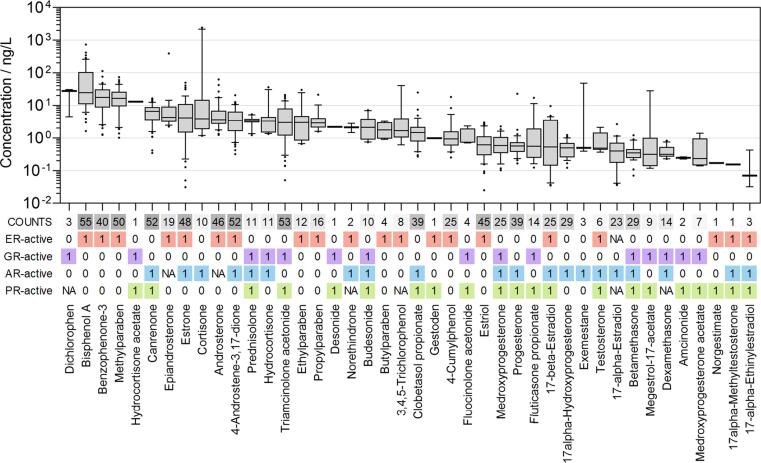
Concentration ranges of the detected chemicals, which were active in at least one GeneBLAzer receptor assay, sorted by the median (zero detects = NA). ‘COUNTS’ represent the number of WWTP effluents, in which the compound was detected. ‘1’ = GeneBLAzer-active compounds, ‘0’ = GeneBLAzer-inactive compounds, ‘NA’ = compounds without single-compound-testing results.

A list of the detected target compounds and the measured mass-based concentrations is provided in the supporting information (Table B5). Of the 79 steroids and phenols analysed, 56 were measured in at least one WWTP effluent sample. This study focused on those compounds which were active towards at least one receptor in the GeneBLAzer assay, which held true for 42 compounds (Fig. 1 and Fig. 4). They form the basis for the following iceberg modelling. Measured concentrations ranged from 25 pg/L (estriol) up to 2.4 μg/L (cortisone). Dichlorophen comprised the highest median concentration (27.7 ng/L) but was only detected in three samples. It was followed by Bisphenol A (24.5 ng/L), which was detected in 55 samples. In total, 13 compounds were detected in more than half of the WWTP effluents, while five compounds were measured only once. Between nine and 23 active target compounds were detected in each WWTP effluent.

Among others, different natural estrogens were detected, including estrone (E1), 17α- and 17β-estradiol (E2) and estriol (E3), as well as the synthetic estrogen 17α-ethinylestradiol (EE2). The highest detected median concentration was found for E1 (4.1 ng/L), which was detected in 48 samples, followed by E3 (0.6 ng/L) in 45 samples and α/β-E2 (0.4–0.5 ng/L) in 23–25 samples, respectively. EE2 was detected in only three samples at concentrations of 0.03, 0.07 and 0.4 ng/L. The synthetic glucocorticoid triamcinolone acetonide was detected in 53 samples with a median concentration of 3 ng/L. A further frequently detected glucocorticoid was betamethasone, detected in 29 samples at a median concentration of 0.3 ng/L. The reference compound of GR-activity, dexamethasone, was detected in 14 samples at a median concentration of 0.3 ng/L. Prominent examples of frequently detected androgens were 4-androstene-3,17-dione (52) and androsterone (46), with median concentrations of 3.4 and 3.6 ng/L, respectively. Testosterone was detected in only six samples at a median concentration of 0.5 ng/L. The highest detected concentration of a common androgen epiandrosterone was 393 ng/L, which was detected in 19 samples. Known progestogens were only detected in a few samples. The reference compound for PR-activity, progesterone, was detected in 39 samples, while megestrol-17-acetate was detected in nine samples, at median concentrations of 0.6 and 0.3 ng/L, respectively. In the following, the results of the chemical analysis are compared with similar studies on treated wastewater samples, focusing on the drivers of the four endpoints investigated (ER, GR, AR and PR).

Estrogen concentrations, i.e. of E1, E2, E3 and EE2, were slightly lower compared to a comprehensive review by Limpiyakorn et al. (2011) on 130 effluent samples from 14 countries. There, average concentrations of 12.7 ng/L, 5.1 ng/L, 6.0 ng/L and 2.4 ng/L were stated, which were detected in 58, 56, 13 and 43 effluent samples, respectively. Lower concentrations in the influents and limited sensitivities of the devices and methods could explain the deviation. The frequently detected compound bisphenol A found in high concentrations (up to 736 ng/L) is a well-investigated contaminant in treated wastewater. Huang et al. (2014) detected a similar median concentration of 78.2 ng/L. The lowest predicted no effect concentration (PNEC) for bisphenol A in freshwater (240 ng/L) is just one order of magnitude higher. In some cases, the detected concentration was even higher than the PNEC, which means existence of regulatory concern. However, for risk assessment, dilution of the effluents with the receiving water would have to be taken into account.

The detected concentrations of the predominant glucocorticoid triamcinolone acetonide (0.05–21 ng/L) agreed well with studies conducted in Germany (5.5–28 ng/L (Weizel et al., 2018)), in the U.S.A (6–14 ng/L (Jia et al., 2016)) and in the Netherlands (14 ng/L (Schriks et al., 2010)). The detected concentrations suggest that the removal of this compound by wastewater treatment is insufficient and that, thus, high amounts of glucocorticoids are released into the aquatic environment via the effluent of wastewater treatment plants. According to Chang et al. (2007), five glucocorticoids, including prednisone, cortisone, cortisol, dexamethasone and 6α-methylprednisolone, were removed by 92–100 % in seven WWTPs, while betamethasone valerate and triamcinolone acetonide were removed by <50 % in laboratory-scale degradation tests with activated sludge after 4 and 24 h, respectively (Miyamoto et al., 2014).

The compounds responsible for androgenic and progestagenic activity, in contrast, are well removed with reported efficiency of up to 91–100 % (Bain et al., 2014, Chang et al., 2008, Houtman et al., 2018), explaining the low detection frequency of related substances. However, AR- and PR-active compounds remain in the studied effluent samples, such as the frequently detected synthetic progestin medroxyprogesterone. In a previous study on effluent samples by Kolodziej et al. (2003), this compound was detected at concentrations up to 15 ng/L. Also, endogenous hormones, including progesterone and testosterone, which are naturally excreted by humans and animals, were detected. According to the literature, both are known to be well removed in wastewater treatment (Chang et al., 2008, Houtman et al., 2018), indicating high loads or poor treatment performance. In sample EU009, an exceptionally high concentration of >2400 ng/L of naturally occurring cortisone was detected. Chang et al. (2007) detected average concentrations at 0.26 ng/L, while Houtman et al. (2018) reported cortisone concentrations above 100 ng/L. In the same sample, synthetic hydrocortisone and gestodene were detected.

### 3.2 Effect-based analysis

#### 3.2.1 Estrogenic risks based on three ERα-assays (EEQ_bio_)

Estrogenicity was detected in 55 (98 %), 42 (75 %) and 49 (88 %) of the 56 WWTP effluent samples for the p-YES, ERα-CALUX® and ERα-GeneBLAzer bioassay (no clean-up), respectively (SI, Table B12). Measured activities were converted into BEQ_bio_, expressed as EEQ_bio_, ranging between 0.01 and 6.3ng_E2_/L (p-YES), 0.05 and 18.5 ng_E2_/L (ERα-CALUX®) and between 0.1 and 8.0 ng_E2_/L (ERα-GeneBLAzer). Six samples were inactive in the ERα-GeneBLAzer but active in the p-YES, with EEQ_p–YES_ between 0.01 and 1.9 ng_E2_/L. 13 samples were inactive in the ERα-CALUX® but active in the p-YES, with EEQ_p–YES_ between 0.01 and 6.3 ng_E2_/L. 15 samples were inactive either in the ERα-GeneBLAzer or in the ERα-CALUX® (eleven and four, respectively). Samples which have received an advanced treatment by AC (EU019) or ozonation (EU032, EU128 and EU130) showed consistently low or no activities, as well as samples EU124 and EU120 (SI, Fig. A5).

**Fig. 2:**
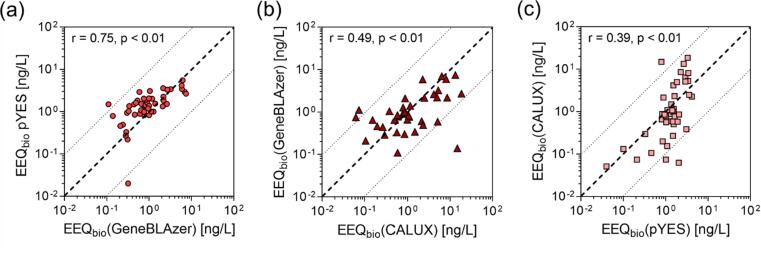
Correlation of the three ERα-assays, incl. r-value from Pearson correlation. (a) ERα-GeneBLAzer vs p-YES, (b) p-YES vs ERα-CALUX® and (c) ERα-CALUX® vs ERα-GeneBLAzer.

The three applied ERα bioassays were compared to each other by Pearson correlation (Fig. 2). The results of the ERα-GeneBLAzer and the p-YES assay were most consistent in terms of a high Pearson correlation (r = 0.75, p < 0.01) and with a small shift towards higher EEQ_p–YES_ (Fig. 2a). For one sample (EU027), the EEQ_p–YES_ was more than one order of magnitude higher than EEQ_GeneBLAzer_. The correlation between EEQ_CALUX_ and EEQ_GeneBLAzer_ was lower (r = 0.49, p < 0.01), while EEQ_p-YES_ and the EEQ_CALUX_ were least consistent (0.39, p < 0.01) (Fig. 2b and Fig. 2c, respectively). For higher EEQ_bio_, there was a shift in the direction of ERα-CALUX®, while for lower values, the correlations shifted towards the other two assays.

However, the three correlation plots consist of different numbers of data points, as different numbers of samples were active in each of the three ERα-assays. Comparing the three assays, only one sample was inactive in the p-YES (EU130), which indicates a high sensitivity of this assay or a higher probability for false-positives. Reasons for the seven inactive samples in ERα-GeneBLAzer could be (i) a lower sensitivity, (ii) masking effects by cytotoxicity (which was not measured in the p-YES) or (iii) the limit of 10 % response (EC_10_) was not reached.

In general, our findings on estrogenic activity align well with previous studies (Leusch et al., 2017) with EEQ_GeneBLAzer_ ranging from 0.11 to 6.5 ng_E2_/L. Medlock Kakaley et al. (2020) also detected a high mean EEQ_bio_ of 15 ng_E2_/L (T47D-KBluc assay) in US secondary stage WWTPs with UV disinfection. Likewise, a study on wastewater in Australia reported mean EEQ_GeneBLAzer_ of effluents from WWTPs with different treatment technologies, which were 27.7 ng_E2_/L (primary stage), 3.2 ng_E2_/L (secondary stage) and 2.0 ng_E2_/L (tertiary stage) (Neale et al., 2020).

#### 3.2.2 Endocrine disruption based on GeneBLAzer assays (BEQ_bio_)

**Fig. 3:**
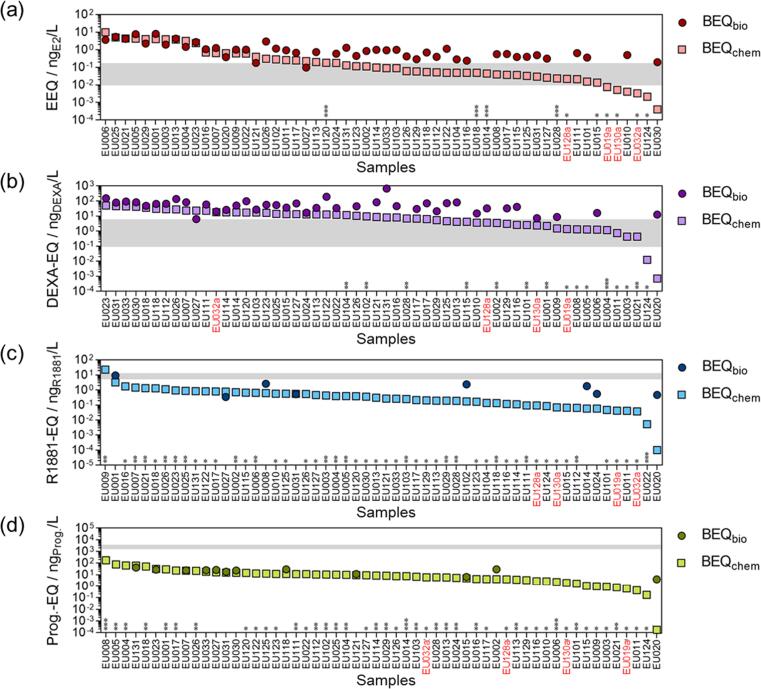
BEQ_chem_ (squares) and BEQ_bio_ (dots) for the GeneBLAzer assays. (a) ER-activity expressed as EEQ, (b) GR-activity expressed as DEXA-EQ, (c) AR-activity expressed as R1881-EQ and (d) PR-activity expressed as Progesterone-EQ. *Not active (negative EC_10_ or EC_10_ > REF100). **Cytotoxicity masked EC_10_. ***Extrapolated EC_10_ (active, but concentration range did not reach the effect level of 10 %). Samples from WWTPs with advanced treatment are highlighted in red (suffix “a”). The grey horizontal belt defines the range in which the EBTs are located according to the three derivation options. (For interpretation of the references to colour in this figure legend, the reader is referred to the web version of this article.)

For a later comparison of the effect-based and chemical analysis (Section 3.3), the derived EC_10_ values from the ERα-, GR-, AR- and PR-GeneBLAzer bioassays (performed on the samples subjected to a previous clean-up) were converted into BEQ_bio_, expressed as EEQ_bio_, DEXA-EQ_bio_, R1881-EQ_bio_ and Progesterone-EQ_bio_, respectively (Fig. 3). The underlying concentration–response curves (CRCs) were listed in the supporting information. An example of the linear portion of the CRC of the ERα-GeneBLAzer assay is shown in the supporting information (Fig. A4).

ERα- and GR-activity was most prominent, detected in 46 (82 %) and 41 (73 %) of all 56 samples, ranging from 0.01 to 8.0 ng_E2_/L and from 6.4 to 676 ng_DEXA_/L, respectively. The latter concentration was by far the highest detected activity in all samples. AR- and PR-activity was detected less often in eight (14 %) and twelve (21 %) samples, respectively. AR-activity ranged from 0.34 to 9.4 ng_R1881_/L, while PR-activity was between 4 and 42 ng_Progesterone_/L. Both were highly affected by cytotoxicity masking effects (** in Fig. 3). In a few cases of WWTP effluent samples, only extrapolated EC_10_ values were available (*** in Fig. 3), which were excluded from the results. In general, sample EU001 showed the highest detected ERα- and AR-GeneBLAzer activity, while sample EU131 was the most active in GR and PR. The blanks did not show any measurable activities in the bioassays.

EEQ_bio_ results of the ERα-GeneBLAzer bioassay were already discussed in the previous section on estrogenicity, noting that the results were largely consistent between non-clean-up and clean-up samples (r = 0.93, p < 0.0001) (SI, Fig. A2). Excluding the highest value of glucocorticogenic activity, the detected range of DEXA-EQ_bio_ (6.4–192 ng_DEXA_/L) is comparable to the results of previous studies, with DEXA-EQ_bio_ of 39–155 ng_DEXA_/L for four secondary stage WWTP effluents in the US (Jia et al., 2016) and DEXA-EQ_bio_ from approx. 39.2 to 300 ng_DEXA_/L in tertiary stage Australian WWTPs (Neale et al., 2020). The sample EU131 yielded the highest glucocorticogenic activity (676 ng_DEXA_/L), also compared to the previously mentioned studies. However, comparisons may be affected by different WWTPs, sampling dates (seasonal impact) and sampling methods. Androgenicity detected in this study (0.47–9.4 ng_R1881_/L) was consistent with or lower than R1881-EQ_bio_ measured downstream of WWTPs, i.a. 3.9 ng_R1881_/L in the Ammer River (Muller et al., 2018) or 5.8 ng_R1881_/L in the Danube River in Novi Sad, Serbia (König et al., 2017). The so-called AR antagonists are able to conceal the agonistic potency of the receptor. In the study of Weiss et al. (2009), the mixture of nonylphenol and dibutyl phthalate contributed to anti-androgenicity, which masked the response of the androgenic compounds in river sediment extracts. Also BPA is a known anti-androgenic compound (Lee et al., 2003). Since nonylphenol and BPA were detected in the present study (among the highest detected median concentrations), we assume a similar situation of masking effects by AR-antagonism. Hence, antagonist mode AR could be an additional reasonable endpoint to the screening of endocrine disruption in WWTP effluents (Mehinto et al., 2015, Shuliakevich et al., 2022). Similar to AR response, progestagenic activity was activated by few of the effluent samples with Progesterone-EQ_bio_ starting from 6.57 ng_progesterone_/L. In some samples, the specific activity was masked by cytotoxicity, others might have been affected by antagonism, which was also described in the EDA study of Hashmi et al. (2020).

#### 3.2.3 Component-based mixture risk assessment based on chemical analysis (BEQ_chem_)

Individual and mixture risks were estimated using BEQ_chem_ based on the specific activities from single compound testing (SI, Table B6). The calculation of BEQ_chem_ was only feasible due to an extensive database of relative effect potency (REP) values for the GeneBLAzer assays, which was expanded for the current study. In principle, BEQ_chem_ could also be calculated for the other assays. However, this would require additional experiments for the p-YES and ERα-CALUX®.

**Fig. 4:**
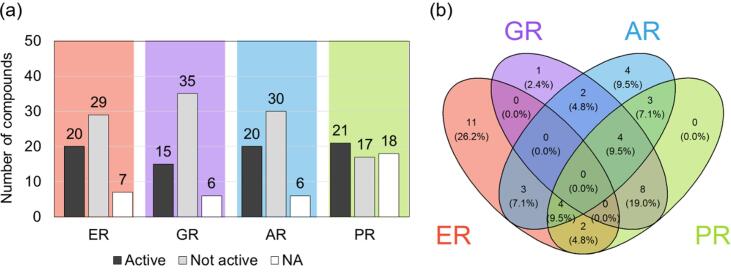
(a) Information on available ERα-, GR-, AR- and PR-GeneBLAzer activity from single-compound testing (for compounds, which were detected in at least one sample). (b) Venn diagram of ER-, GR, AR and PR-GeneBLAzer-active compounds.

From the list of detected compounds, 20, 15, 20 and 21 chemicals were active in ERα-, GR-, AR- and PR-GeneBLAzer, respectively, forming the basis of iceberg modelling (Fig. 4a). In total, 29, 35, 30 and 17 chemicals, respectively, were considered not active. For seven, six, six and 18 compounds, respectively, the required effect data was not available. Of the compounds linked with estrogenicity, eleven were active in ERα only, while three, two and four were also AR- or PR- active or both, respectively (Fig. 4b). GR-active compounds overlapped with PR-active compounds in eight cases, and four compounds were active towards all receptors except ERα.

Predicted mixture effects based on measured concentrations of active steroids and phenols are shown in Fig. 3 and range from 0.4 pg_E2_/L to 10 ng_E2_/L for ER-activity, 0.7 pg_DEXA_/L–50 ng_DEXA_/L for GR-activity, 0.1 pg_R1881_/L–23 ng_R1881_/L for AR-activity and 0.2 pg_Progesterone_/L–175 ng_Progesterone_/L for PR-activity (SI, Table B7). The contribution of each chemical (BEQ_chem,*i*_) to BEQ_chem_ and the number of active chemicals per sample and assay are shown in the SI (Figures A6-9). For ER-active samples, the compound dominating the BEQ_chem_ was E2, followed by E1, E3 and EE2 and bisphenol A (SI, Fig. A6). Between four and 14 active compounds per sample (EU130 and EU001, respectively) contributed to the predicted ER-activity. Certain compounds with comparably high detected concentrations and low receptor-mediated specific activity were only minor contributors of BEQ_chem_, as for example the paraben xenoestrogens, which are several orders of magnitude less potent (lower REP) than EE2. BEQ_chem_ of GR-active samples were dominated by triamcinolone acetonide (SI, Fig. A7). Further compounds active towards GR and with higher contributions in single samples were fluocinolone acetonide, fluticasone propionate, budesonide and desonide, while a maximum of seven compounds contributed in one sample (EU127). AR-activity was mainly explained by cortisone, progesterone, medroxyprogesterone, testosterone and hydrocortisone (SI, Fig. A8). The sample with the highest number of 13 active compounds was the same as previously for GR (EU127). The main contributors to PR-activity were clobetasol propionate and medroxyprogesterone acetate, as well as gestoden, megestrol-17-acetate and progesterone but only in few samples (SI, Fig. A9). The highest number of active compounds was ten (EU006 and EU007).

### 3.3 Iceberg Modelling: Linking chemical and effect-based analysis

Results of the receptor-mediated GeneBLAzer assay and the chemical target analysis were compared by iceberg modelling based on the measured and predicted bioanalytical equivalent concentrations (BEQ_bio_ vs BEQ_chem_), separately for each receptor (Fig. 3). For calculating BEQ_chem_ the extensive database of single substance activities measured in the GeneBLAzer bioassay (EC_10,*i*_) was used. Taking EC_10,*i*_ from the GeneBLAzer assay in order to calculate BEQ_chem_ for ERα-CALUX® or p-YES is not feasible due to different REP_*i*_ values (Escher et al., 2021).

**Fig. 5:**
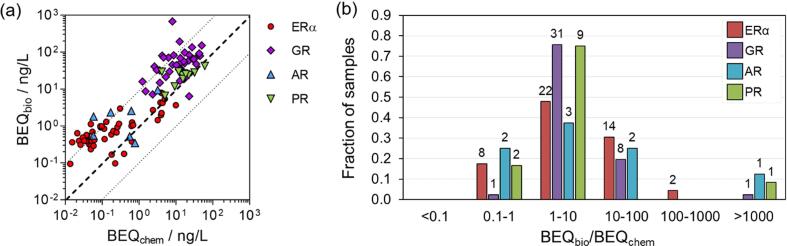
(a) Correlation of BEQ_bio_ vs BEQ_chem_ (Iceberg Modelling). Concentrations of compounds detected in at least one WWTP effluent sample, which were not available (NA) were set to zero. (b) Distribution of BEQ_bio_/BEQ_chem_-ratios, showing the fraction of samples per ratio category relative to the absolute number of active samples (ERa-active = 46, GR-active = 41, AR-active = 8, PR-active = 12). The absolute number of samples per ratio category and assay is indicated as a number above each bar.

If the results of the ERα-, GR-, AR- and PR-GeneBLAzer assay were explained entirely by the results of the chemical target analysis, the outcome would be a one-to-one correlation for all four endpoints (Fig. 5a, dashed line). For most active samples, the ratio of BEQ_bio_ and BEQ_chem_ was between 1 and 10 (Fig. 5b), and thus above the one-to-one correlation. Deviations from the one-to-one correlation may be due to analytical and bioanalytical inaccuracies. The BEQ_bio_/BEQ_chem_-ratios lower than 1 found in eight, one, two and two samples for ERα, GR, AR and PR, respectively, may originate from the presence of antagonists supressing BEQ_bio_, as discussed for anti-androgenic effects of nonylphenol, dibutyl phthalate and BPA. In most cases BEQ_bio_/BEQ_chem_-ratios were up to one order of magnitude greater than 1 indicating (i) missing compounds in our target list or (ii) non-detects due to chemical concentrations below the method detection limit (MDL) (Könemann et al., 2018). Assuming concentrations of non-detects of MDL/2 or MDL significantly shifts the ratio towards the one-to-one correlation for all endocrine disruptors except for PR-active compounds (SI, Fig. A10b and A10c). Thus, it may be hypothesized that ERα-, GR- and AR-active steroids might be frequently present at concentrations below but close to the MDL, while an assumption of MDL for PR-active compounds substantially overestimates the risk (SI, Fig. A10b).

### 3.4 Assessment of the endocrine disruptive potential of WWTP effluents

#### 3.4.1 Assessment of EEQ_bio_ against literature thresholds

**Fig. 6:**
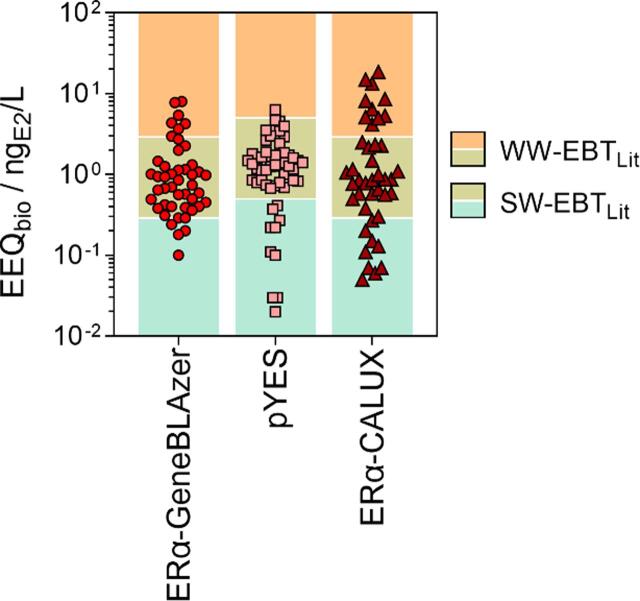
Measured EEQ_bio_ for the three estrogenicity bioassays, incl. mean EBT-EEQ values according to the literature (Brion et al., 2019, Escher et al., 2018a, Jarosova et al., 2014, Kunz et al., 2015, van der Oost et al., 2017). SW-EBT_Lit_: Threshold for surface water, WW-EBT_Lit_: Threshold for wastewater, incl. a dilution factor of 10.

Measured values for EEQ_bio_ were compared to effect-based trigger values (EBTs) to differentiate between poor and acceptable water quality. Unfortunately, there are no bioassay-specific EBTs in regulation, but only preliminary values used in the research context. According to different studies on estrogenicity in surface water, EBT-EEQs for the ERα-GeneBLAzer, the p-YES and the ERα-CALUX® bioassays ranged between 0.1 and 0.5 ng_E2_/L (Brion et al., 2019, Escher et al., 2018a, Jarosova et al., 2014, Kunz et al., 2015, van der Oost et al., 2017). Taking the mean EBT-EEQ derived from literature values per bioassay and for surface water (SW-EBT_Lit_) allowed a first assessment of the water quality according to the three bioassays. The SW-EBT_Lit_ were 0.29, 0.5 and 0.29 ng_E2_/L for ERα-GeneBLAzer, p-YES and ERα-CALUX®, respectively (Fig. 6). There were 40, 44 and 33 ERα-active samples, respectively, exceeding the bioassay-specific thresholds.

Acknowledging that 100 % wastewater in a river is a worst-case scenario and assuming typical dilution factors of WWTP effluents in small rivers of 1 to 10 (realistic best-case scenario), we use both scenarios for the evaluation of EEQ_bio_: SW-EBT_Lit_ (no dilution) as the lower limit and wastewater-specific EBTs (WW-EBT_Lit_, 10fold dilution) as the upper limit (Fig. 6). Applying WW-EBT_Lit_ values, seven, one and ten samples would still exceed the threshold, respectively, indicating poor and unacceptable water quality in the corresponding cases.

#### 3.4.2 Assessment of BEQ_bio_ and BEQ_chem_ against tentative thresholds

EBTs are increasingly used for research purposes, as shown by the previous example on estrogenicity. However, for other receptor-based bioassays, such as the GR-, AR- and PR-GeneBLAzer assay, only a few or no information on EBTs are available in the literature. This is due to the lack of guideline values for chemicals triggering these effects and the wide range of REPs of agonists of these receptors. Different derivation options have been discussed (Escher et al., 2018a), of which two were applied in this study and introduced in the methods section of this paper (Table 1, “Core equation” and “Option 1”). By translating EQS values (*in vivo* effects) into their corresponding compound specific EBT_*i*_ values (*in vitro* effects), as described in the methods section, we would be able to account for differences in potency between *in vivo* and *in vitro* effects (Table 1, “Option 2” and “Option 3”). This would allow for the derivation of EBTs using validated EQS and PNEC values (for the calculation of REP_*in vivo*_ according to Equation (4)), which are supposed to be protective for organisms sensitive to the specific chemicals but not availabe for all compounds. Still, the available information was sufficient to define preliminary EBT ranges (Fig. 3, grey horizontal belt), which in the following were applied to the measured and predicted effects (BEQ_bio_ and BEQ_chem_). Dilution of wastewater discharges in the surface water was not considered in this approach. More detailed information on the derivation of EBTs can be found in the supporting information (Section A2.4).

Endpoint-specific EBTs according to the different options ranged roughly within one order of magnitude reflected by the grey horizontal belt (Fig. 3a–d). EEQ_bio_ of all samples except two would exceed the EBT, while EEQ_chem_ would exceed the upper limit in 39 % and the lower limit in 89 % of the samples (Fig. 3a). Also for DEXA-EQ_bio_ most samples exceeded the range (96 %), while for DEXA-EQ_chem_ only two samples fell below the range (Fig. 3b). In the case of R1881-EQ_chem_ and R1881-EQ_bio_ most samples (98 %) were below the EBT or not active. Since only eight samples exhibited measurable effects this result might be not very robust (Fig. 3c). Both for Progesterone-EQ_bio_ and Progesterone-EQ_chem_, no cases of EBT exceedance were found (Fig. 3d).

It should be stressed here, that missing target compounds and non-detects could have lowered the BEQ_chem_, as discussed previously (Section 3.3), which is why the application of assay-related EBT values on BEQ_chem_ has to be taken with care. Furthermore, the EBTs derived for this study are only preliminary based on the limited data available. Thus, there is a strong need to enhance the availability of experimental and monitoring-based PNEC_*i*_ values to calculate more robust EBT values. However, the results provide first evidence that at least for ER- and GR-active compounds many WWTP effluents exceed levels of concern.

#### 3.4.3 Impact of advanced treatment technologies

Samples, taken after an advanced treatment by ozonation (EU032, EU128 and EU130) and activated carbon (EU019) showed a generally low number of detected compounds (12–16) along with a low total sum of single measured concentrations (SI, Fig. A3). The same observation was made for a set of 366 further emerging pollutants for the same dataset (Finckh et al., 2022). When looking at the three WWTPs equipped with ozonation, where samples were taken before and after the additional treatment step (EU031 vs EU032, EU127 vs EU128 and EU129 vs EU130), ER-activity was removed and GR-activity was reduced in all three cases (79 vs 20 ng_DEXA_/L, 69 vs 32 ng_DEXA_/L and 33 vs 7.2 ng_DEXA_/L, respectively) (SI, Table B7). Regarding GR-activity, highly variable removal efficiency between −7 and 100 % were reported (Bain et al., 2014, Houtman et al., 2018, Neale et al., 2020, Roberts et al., 2015). The application of ozone was shown to remove GR activity significantly, but only at relatively high dose compared to the total organic carbon of the water (ozone:TOC of 1:1) (Jia et al., 2016). In previous studies on ER-activity in treated wastewater, removal efficiencies by oxidation of more than 95 % for E1, E2, E3 and EE2 were achieved (Deborde et al., 2005, Nazari and Suja, 2016). Also a recent study by Wolf et al. (2022) indicated the nearly fully elimination of estrogenic potential after ozone treatment. However, also a significant impact of a rain overflow basin (ROB) located upstream of the investigated WWTP effluent was shown. The highest endocrine potential was found after the ROB overflow (2.7 ng_E2_/L), indicating that heavy rainfall and runoff events have a large impact on the endocrine load of the receiving waters.

## 4 Conclusion

Estrogenic, androgenic, glucocorticoid and progestagenic compounds were detected in WWTP effluents by chemical and effect-based methods supporting that EDCs enter the environment via treated wastewater. Common steroids were detected frequently, such as estrone (E1), bisphenol A, triamcinolone acetonide, medroxyprogesterone and clobetasol propionate. Most effluent samples were active in the ERα- and GR-GeneBLAzer assays (82 % and 73 %), but only few in AR- and PR-GeneBLAzer assays (14 % and 21 %). The main contributors of PR-activity were successfully identified. At the same time, insufficient method detection limits, missing compounds in our target list and the presence of antagonists could be reasons for mismatches of BEQ_chem_ and BEQ_bio_ in the case of ER-, GR- and AR-activities. As shown for estrogenicity, effect-based results (EEQ_bio_) depend on the applied assay. While we have found a good correlation of the results from the GeneBLAzer and the p-YES (r = 0.75), the CALUX® correlated only with r = 0.49 and r = 0.39, respectively.

There is an urgent need to further develop effect-based trigger values (EBTs) in order to assess the endocrine disruptive potential of effluents and to provide an estimate of poor and acceptable water quality. Tentative trigger values, which were applied on both measured and predicted results (BEQ_bio_ and BEQ_chem_, respectively), indicate rather poor water quality for nearly all samples, according to at least one and up to three endpoints (ER, GR and AR). While many of the major effect-drivers are hardly removed by conventional treatment plants, ozonation and activated carbon treatment can help reduce EDC contamination in the aquatic ecosystem. Samples from WWTPs with such additional treatment were only active in GR, with reduced activities comparing samples from before and after the ozonation treatment.

In conclusion, the comparison of three different receptor-based bioassays for ERα, the use of GR-, AR- and PR-GeneBLAzer bioassays and the investigation of various EBTs contributed to a better understanding of the potential of using bioassays for scientific but also regulatory purposes. As shown in the present study on European WWTP effluents, future investigations and assessments of water quality can be highly improved by linking chemical with effect-based analytical tools.

## CRediT authorship contribution statement

**Saskia Finckh:** Conceptualization, Validation, Formal analysis, Investigation, Data curation, Writing – original draft, Writing – review & editing, Visualization, Supervision, Project administration. **Sebastian Buchinger:** Conceptualization, Validation, Investigation, Resources, Writing – review & editing, Funding acquisition. **Beate I. Escher:** Conceptualization, Methodology, Validation, Formal analysis, Resources, Data curation, Writing – review & editing, Supervision, Funding acquisition. **Henner Hollert:** Conceptualization, Validation, Resources, Writing – review & editing, Funding acquisition. **Maria König:** Validation, Investigation, Writing – review & editing. **Martin Krauss:** Conceptualization, Methodology, Software, Validation, Investigation, Data curation, Writing – review & editing, Supervision. **Warich Leekitratanapisan:** Conceptualization, Validation, Formal analysis, Investigation, Data curation, Writing – original draft, Writing – review & editing, Visualization. **Sabrina Schiwy:** Conceptualization, Validation, Writing – review & editing. **Rita Schlichting:** Software, Validation, Formal analysis, Data curation, Writing – review & editing. **Aliaksandra Shuliakevich:** Conceptualization, Validation, Formal analysis, Investigation, Writing – review & editing. **Werner Brack:** Conceptualization, Validation, Resources, Writing – original draft, Writing – review & editing, Supervision, Funding acquisition.

## Declaration of Competing Interest

The authors declare that they have no known competing financial interests or personal relationships that could have appeared to influence the work reported in this paper.

## Data Availability

Data will be made available on request.
