# Supplementary material for: Endocrine disrupting chemicals entering European rivers: Occurrence and adverse mixture effects in treated wastewater
Source: Environ Int. 2022 Dec;170:107608. doi: 10.1016/j.envint.2022.107608 (PMC9720157; doi:10.1016/j.envint.2022.107608)
Supplement: Appendix A [file mmc1.docx]

Supporting Information

A

**Endocrine disrupting chemicals entering European rivers: Occurrence and adverse mixture effects in treated wastewater**

Saskia Finckh^1,2^, Sebastian Buchinger^3^, Beate I. Escher^4,5^, Henner Hollert^2^, Maria König^4^, Martin Krauss^1^, Warich Leekitratanapisan^6^, Sabrina Schiwy^2^, Rita Schlichting^4^, Aliaksandra Shuliakevich^2^, Werner Brack^1,2^

^1^ Department of Effect-Directed Analysis, UFZ – Helmholtz Centre for Environmental Research, Leipzig, Germany.

^2^ Department of Evolutionary Ecology and Environmental Toxicology, Goethe University, Frankfurt am Main, Germany.

^3^ Department of Biochemistry and Ecotoxicology, Federal Institute for Hydrology – BfG, Koblenz, Germany.

^4^ Department of Cell Toxicology, UFZ – Helmholtz Centre for Environmental Research, Leipzig, Germany.

^5^ Environmental Toxicology, Department of Geosciences, Eberhard Karls University, Tübingen, Germany.

^6^ Environmental Toxicology Unit – GhEnToxLab, Faculty of Bioscience Engineering, Ghent University, Ghent, Belgium.

E-mail of corresponding author: saskia.finckh@ufz.de

# Material and Methods

## Sample processing – additional information

### Clean-up of samples using an aminopropyl column

The clean-up of the samples prior to the analysis was similar for the chemical and effect-based analyses, except for (i) the addition of internal standards and (ii) the applied volumes. First, the aminopropyl columns (3 mL/500 mg, Sigma-Aldrich SupelClean LC-NH2) were conditioned with ethyl acetate, followed by ethyl acetate:methanol 80:20. An aliquot of the LVSPE extracts (REF 1000) and ethyl acetate were transferred into a 2 mL autosampler vial (Phenomenex). For chemical analysis, 10 µL of internal standard mixture (1 µg/mL) were added. For effect-based analysis (GeneBLAzer assays) this step was skipped, that is, no internal standard mixture was added. Next, the sample was applied to the pre-conditioned column, the autosampler vial rinsed twice with ethyl acetate:methanol 80:20 (0.25 mL) and the rinse volume added to the column. After elution with ethyl acetate:methanol 80:20 into a 7 mL vial, the solvent was evaporated with a gentle nitrogen stream and the sample re-dissolved in methanol. The applied volumes according to the different purposes (chemical or effect-based analysis) are listed in Table A1. For the chemical analysis, the reconstituted sample was divided into two aliquots (2x250 µL in 2 mL autosampler vials), one for further derivatisation with dansyl chloride (“aliquot 2”, see section below) and the other for LC-MS/MS analysis (“aliquot 1” in vial with insert). Subsequently, both aliquots were dried completely using a gentle stream of nitrogen at room temperature.

Table A1: Overview of the aminopropyl clean-up and the applied volumes of solvents and solvent mixtures.

| Processing step | Chemical analysis | Effect-based analysis |
| --- | --- | --- |
| Conditioning | 3 mL EtAc  3 mL EtAc:MeOH (80:20) | 5 mL EtAc  5 mL EtAc:MeOH (80:20) |
| Sample preparation | 200 μL LVSPE extract  800 μL EtAc  10 μL internal standard mix | 1 mL LVSPE extract  4 mL EtAc |
| Extraction | 2 mL EtAc:MeOH (80:20) (2x) | 2 mL EtAc:MeOH (80:20) |
| Reconstitution | 500 μL in MeOH (2x 250 µL) | 1 mL in MeOH |
| Measurements | Aliquot 1: LC-MS/MS  Aliquot 2: LC-HRMS (incl. derivatisation) | ERα-, GR-, AR- and PR-GeneBLAzer Assay |

### Derivatisation of estrogens and phenols using dansyl chloride

For the derivatisation of estrogens and phenols, 50 mM NaHCO_3_ solution (Merck, 200 µL) was added to aliquot 2, followed by 0.5 mg/mL dansyl chloride solution (Honeywell, 200 µL). After each addition step, the samples were vortexed (15 s). The samples were stored at 60°C for 10 min and MTBE (Honeywell, 1 mL) was added. Afterwards, the samples were again vortexted (1 min) and put into a freezer ($-$20°C) for 30 min to support phase separation. The complete MTBE phase was withdrawn with a Pasteur pipette and transferred into a 2 mL autosampler vial with conical bottom. The MTBE phase was dried completely using a gentle stream of nitrogen at room temperature and stored at $-$25°C. Prior to LC-HRMS analysis the samples were reconstituted in methanol:water 70:30 (100 µL).

## Chemical target analysis – additional information

### Method-matched calibration standards

The calibration standards for the chemical target analysis were prepared based on stock solutions of more than 40 steroids (S) and 30 phenols (P) according to Table A2. All standards were processed according to the previously introduced clean-up and derivatization procedure.

Table A2: Overview of applied volumes for the preparation of calibration standards.

| Volumes / µL | Standard Mix 1 µg/mL | Standard Mix 20 µg/mL | Standard Mix 0.5 µg/mL | IS Mix 1 µg/mL | MeOH | EtAc |
| --- | --- | --- | --- | --- | --- | --- |
| Calib Blank | - | - | - | 10 | 200 | 800 |
| Calib P 1000/S 100 ng/mL | 200 | - | - | 10 | 0 | 800 |
| Calib P 500/S 50 ng/mL | 100 | - | - | 10 | 100 | 800 |
| Calib P 200/S 20 ng/mL | 40 | - | - | 10 | 160 | 800 |
| Calib P 100/S 10 ng/mL | 20 | - | - | 10 | 180 | 800 |
| Calib P 50/S 5 ng/mL | 10 |  | - | 10 | 190 | 800 |
| Calib P 20/S 2 ng/mL | - | 200 | - | 10 | 0 | 800 |
| Calib P 10/S 1 ng/mL | - | 100 | - | 10 | 100 | 800 |
| Calib P 5/S 0.5 ng/mL | - | 50 | - | 10 | 150 | 800 |
| Calib P 2/S 0.2 ng/mL | - | 20 |  | 10 | 180 | 800 |
| Calib P 1/S 0.1 ng/mL | - | 10 |  | 10 | 190 | 800 |
| Calib P 0.5/S 0.05 ng/mL | - | - | 200 | 10 | 0 | 800 |
| Calib P 0.2/S 0.02 ng/mL | - | - | 80 | 10 | 120 | 800 |
| Calib P 0.1/S 0.01 ng/mL | - | - | 40 | 10 | 160 | 800 |

### LC-MS/MS analysis

For LC-MS/MS analysis of ketosteroids and phenols including all bisphenols, aliquot 1 (LVSPE extract after aminopropyl clean-up) was used. A sample volume of 5 µL was injected into an Agilent 1260 LC system coupled to an ABSciex QTrap 6500 MS. The compound quantification was accomplished using the vendor software MultiQuant 3.0 (ABSciex).

Ketosteroids were separated by gradient elution with water (eluent A) and methanol (eluent B), each containing 0.1% of formic acid, using a Kinetex C18 column (100x3, 2.6 μm, Phenomenex). The elution was started at 40% B (1 min) and increased to 95% B (within 10.1 min). In the end, the flow was re-equilibrated back to 40% B (within 2 min). For the ketosteroid analysis, the mass spectrometer was operated in positive mode electrospray (ESI$+$). Phenols were separated by gradient elution with water (eluent A) and methanol (eluent B), each containing 1 mM NH4F, using a Kinetex XB-C18 column (100x3, 2.6 μm 100A). The gradient started at 30% A and increased to 95% B (within 12 min). Finally, the flow was re-equilibrated back to 30% A (2.7 min). The mass spectrometer was operated in negative mode (ESI$-$). For both, ketosteropid and phenol analysis, two transitions were measured for each compound in multiple reaction monitoring (MRM) mode.

### LC-HRMS analysis

For LC-HRMS analysis of estrogens and some phenols, aliquot 2 (LVSPE extract after aminopropyl clean-up and dansyl chloride derivatisation) was used. A sample volume of 10 µL was injected into an Ultimate 3000 LC system coupled to a Thermo QExactive MS. The compound quantification was accomplished using the vendor software TraceFinder 4.1 (Thermo Scientific).

Estrogens and phenols were separated by gradient elution with water containing 1 mM ammonium fluoride (NH_4_F) (eluent A) and methanol (eluent B), using a Thermo Phenyl-hexyl column (100x3 mm, 2.6 μm) with a pre-column (4x2.1 mm) and an in-line filter (0.2 μm). The column temperature was 40°C. The elution started at 45% B and 55% A (1 min), then increased linearly to 85% B (within 6 min), and further increased to 100% B (within 18.1 min). In the end, the column was cleaned by flushing with 90% isopropanol, 5% A and 5% B (1.1 min), and re-equilibrated to the initial conditions (2.8 min). The mass spectrometer was operated in ESI$+$ full scan mode with a resolving power of 35,000.

### QA/QC summary of the chemical analysis

For quantification, a 13-point method-matched calibration curve of the target compounds (SI, Table B4) and an internal standard mixture of 39 isotope-labelled compounds (SI, Table B3) were used. Solvent blanks were injected every 10 samples, where no column carryover was detected. Two calibration standards (e.g. 5 μg/L and 20 μg/L) were injected every 20 samples (i.e. at the end of each sequence) to monitor mass accuracy, intensity changes and as a quality control during peak picking. Processing blanks (LC-MS grade water through SPE) were analysed alongside the samples, to exclude contamination by the solvents and materials used.

## Effect-based analysis – additional information

### P-YES bioassay


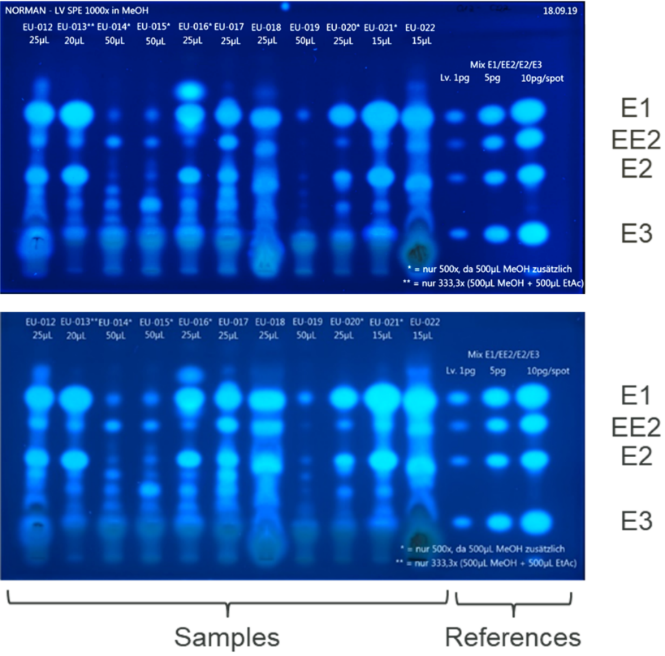


Figure A1: Two replicate plates as examples of the p-YES test.

One day prior to the performance of the p-YES 20 mL growth medium (Yeast Nitrogen Base 6.7 g/L, α-D-Glucose 20 g/L, L-Lysine 36 mg/L, L-Histidine 24 mg/L) were inoculated with 1 mL of a cryo-stock. Cells were harvested after an overnight growth of 22 ± 1 h at 30°C on a darkened shaker (IKA^®^KS 3000 i control, orbital shaking at 200 rpm) by a centrifugation at 2500 g and adjusted to a cell density of 8 x 107 cells/mL ± 10% (FAU = 1500) by resuspension in exposure medium (Yeast Nitrogen Base 31.4 g/L, α-D-Glucose 138.8 g/L, L-Lysine 168.8 mg/L, L-Histidine 112.5 mg/L, Ampicillin sodium salt 375 mg/L, Streptomycin sodium salt 375 mg/L, CuSO4 x 5 H_2_O 35 mg/L). The yeast cells were applied on the HPTLC-plate by spraying (Schoenborn et al., 2017). 3 mL of the yeast cell suspension was sprayed on the dried HPTLC-plate using a derivatizer (CAMAG, Muttenz, yellow nozzle at spraying mode 5). Subsequently, the plates were incubated at 30°C for 3 h in a saturated humid atmosphere (NuAire CO2-incubator with humidity control NU-5820E, at > 90% relative humidity). After the incubation 2.5 mL lacZ-buffer (Na_2_HPO_4_ x 2 H_2_O 10.67 g/L, KCL 0.75 g/L, MgSO_4_ x 7 H_2_O 0.25 g/L, NaH_2_PO_4_ 5.5 g/L, pH = 7; sodium dodecyl sulphate 1 g/L) containing 0.5 mg/mL 4‑methylumbelliferyl-β-D-galactopyranoside (MUG, Sigma Aldrich) were sprayed on the dried HPTLC-plate (cold air-blow, 3-5 min) using a derivatizer (CAMAG, Muttenz, green nozzle at spraying mode 5). For the cleavage of MUG to produce the fluorescent 4-methylumbelliferone the plates were finally incubated at 37°C for 15 min. The fluorescent signals were detected at λ_ex_ = 366 nm with a TLC Visualizer (CAMAG, Muttenz). In addition to visual and photographic detection, fluorescence measurement was performed by using a TLC Scanner 4 (CAMAG, Muttenz) with λex = 320 nm (deuterium lamp), cut-off filter of 440 nm and a slit dimension of 6 x 0.3 mm.

### GeneBLAzer assay

For the assay a defined amount of cells (ERa 5000 cells/well, AR 6500 cells/well, GR 6000 cells/well, PR 5250 cells/well ) were seeded per well. Therefore 30 µL of the cell suspension was added to every well of a 384 Poly-D-Lysine coated, black well plate with clear bottom (Corning BV Life Sciences, # 354663) by using a microplate cell dispenser (MultiFlo, Biotek). In the last row just 30 µL assay medium was added as cell-free control. The cell plate was incubated for 24 h at 37 °C and 5% CO2 before exposing with the samples. 10 µL of the sample were added to the cell plate and the cells were incubated for further 24 h at 37 ° and 5% CO2. The cell confluency was measured by using the cell imagr Incucyte S3 (Biotek) after 24 h and 48 h. At last, 8 µL of the FRET substrate were added according to the manufacturer and the fluorescence was measured using a Tecan M1000 Pro reader.

Table A3: Overview on the performed GeneBLAzer assays.

| Procedure | ERα-GeneBLAzer | GR-GeneBLAzer | AR-GeneBLAzer | PR-GeneBLAzer |
| --- | --- | --- | --- | --- |
| **Seeding** |  |  |  |  |
| Cell line (ThermoFisher) | Erα-UAS-*bla* GripTite Cells | GR-UAS-*bla* HEK 293T Cells | AR-UAS-*bla* GripTite Cells | PR-UAS-*bla* HEK 293T Cells |
| Seeding density / cells/well | 5000 | 5000 | 5000 | 5020 |
| Seeding volume / μL/well | 30 | 30 | 30 | 30 |
| **Dosing** |  |  |  |  |
| Sample dosing / REF | 0.1-100 | 0.1-100 | 0.1-100 | 0.1-100 |
| Reference compounds | 17β-estradiol | Dexamethasone | R1881 | Progesterone |
| concentration (min-max) / nM) | (0.0001-14) | (0.0015-49) | (0.0013-142) | (0.012-635) |
| Dosing volume / μL/well | 10 | 10 | 10 | 10 |
| **Response detection** |  |  |  |  |
| Cytotoxicity assessment | Cell viability detected from IncuCyte t0 (directly after dosing) and t24 (24 h after dosing) | | | |
| Receptor mediated response | Detection with FRET fluorescence at t0h (directly after addition of FRET substrate) and t2h (2 h after addition of FRET substrate) | | | |

### QA/QC summary of the effect-based analysis

Different QA/QC measures were taken for the effect-based analyses as mentioned in the methods sections of each bioassay and summarised in Table A4. Samples analysed in the p-YES and ERα-CALUX^®^ assay were not subjected to a clean-up, while the GeneBLAzer assay was performed twice: once with the raw SPE extract, and once with the extract subjected to a clean-up via an aminopropyl column (Figure A1a). With the exception of three samples (EU027, EU113 and EU129) the EEQ were within a factor of two between the samples with and without clean-up of the SPE extract and there was no systematic deviation (Figure A1b).

It is common practise to test SPE extracts directly, i.e, without additional clean-up step in *in-vitro* bioassays, while chemical analysis requires the additional clean-up to achieve better detection limits. Since only the data of the GeneBLAzer assays were used for Iceberg Modelling (i.e. linking the predicted mixture effects from chemical analysis with the bioassay results), only the GeneBLAzer assays were performed on both types of extracts, with and without clean-up.

Table A4: QA/QC summary of all performed bioassays.

| QA/QC | p-YES | ERα-CALUX^®^ | GeneBLAzer |
| --- | --- | --- | --- |
| Cell viability | NA | Cytotoxicity testing by MTT assay (Mosmann, 1983) | Cytotoxicity testing by IncuCyte at t_0_ (directly after dosing) and at t_24_ (24 h after dosing) |
| Replicates | 2 replicates (deviation range given in Table B12) | 2-4 replicates (standard deviation given in Table B12 | No independent replicates but 11-point duplicate concentration-response curve (standard deviation given in Table B12) |
|  |  |  |  |
| Processing blanks (LC-MS grade water through SPE) | Not active | Not active | Not active |
| Negative controls (blanks) | Not active | Not active (unexposed cells in assay medium) | Not active (unexposed cells in assay medium) |
| Positive controls | E1, E2, EE2, E3 | E2 | E2, dexamethasome, R1881, |
| Concentration response curve (CRC) of reference compound | 3-point CRC of E2 | 9-point CRC of E2 | 11-point CRC of E2 (ERα), dexamethasone (GR), R1881 (AR) and Progesterone (PR) |
| Verification of assay performance | Inter-assay controls by characterization of 5-point CRC on regular basis | Regular inter-assay controls | Regular inter-assay controls and long-term records with Shewart plots |


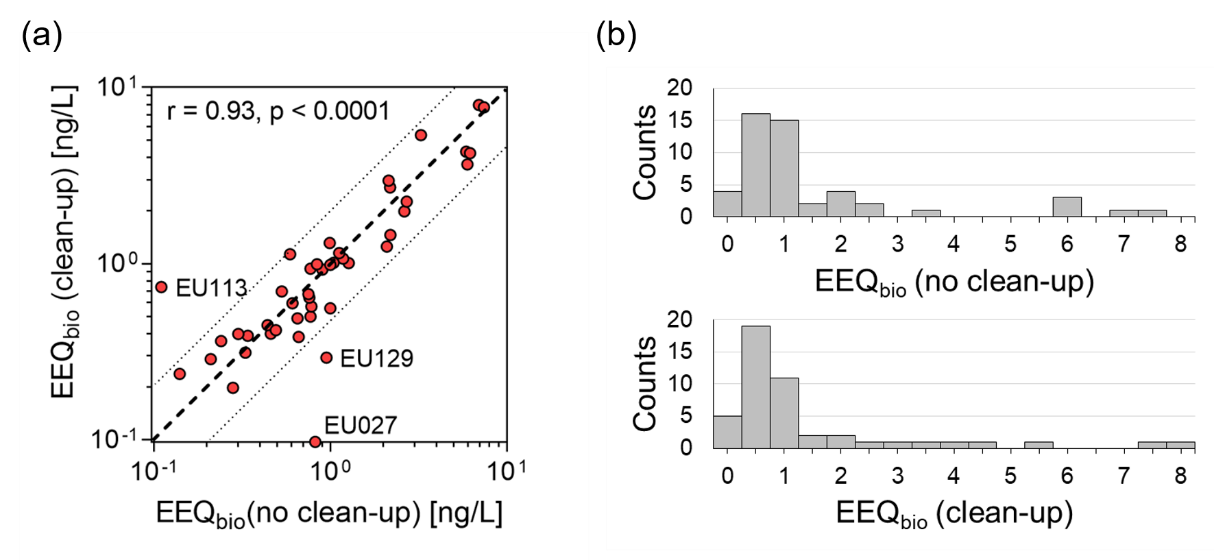


Figure A2: (a) Comparison of non-clean-up and clean-up samples analysed in the ERα-GeneBLAzer assay. (b) Histograms of EEQ_bio_ of non-clean-up and clean-up samples in the ERα-GeneBLAzer assay.

# Results and Discussion

## Chemical target analysis


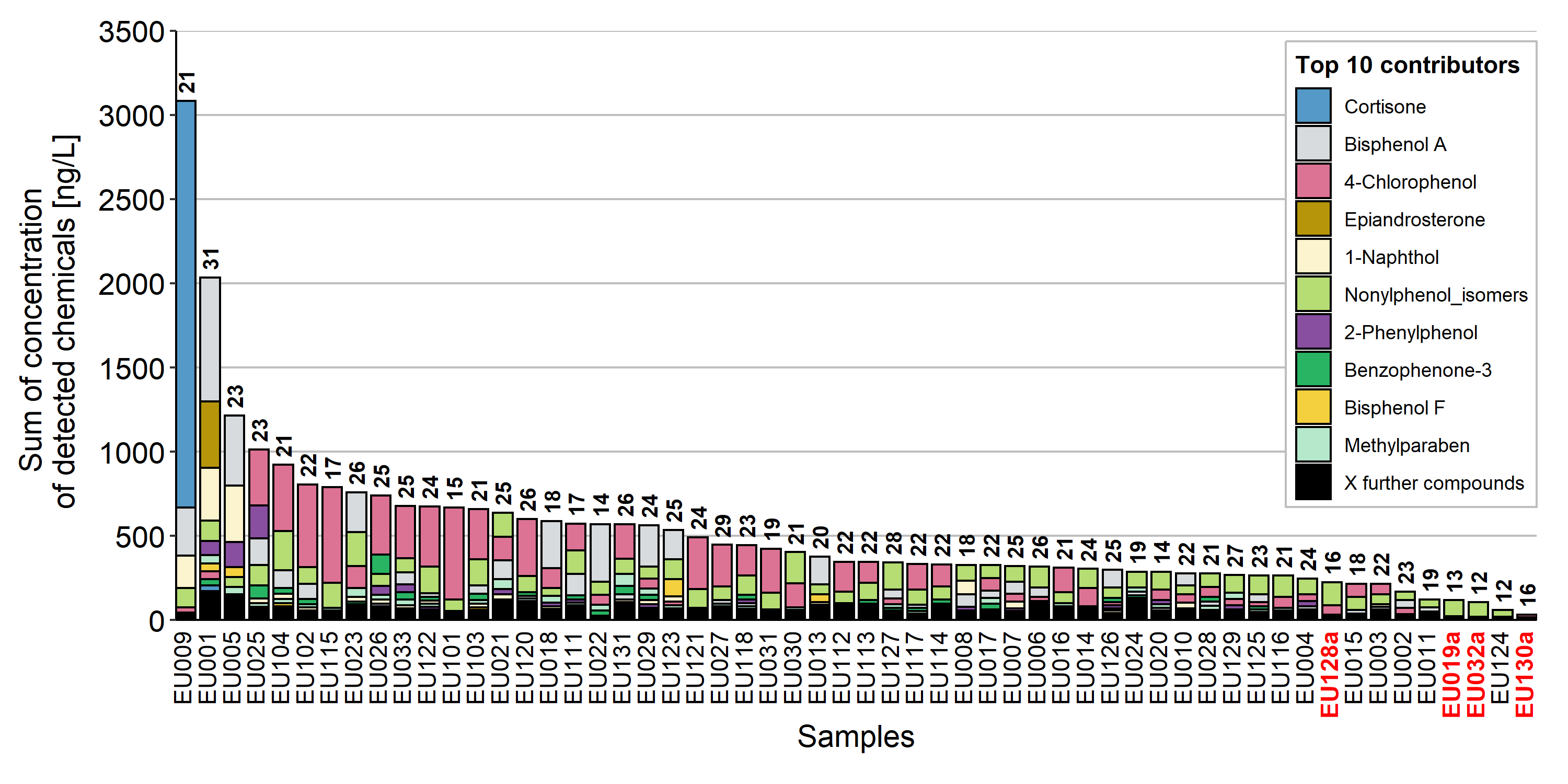


Figure A3: Sum of mass-based concentrations of the detected chemicals (bars), and number of detected chemicals (number above each bar). The coloured stacks within the bars represent the individual concentrations of the top 10 contributors by maximum concentration (over all samples), and are sorted by increasing values. Further compounds are aggregated in the black coloured stacks at the bottom of each bar (“X further compounds”). Samples from WWTPs with advanced treatment are highlighted in red (suffix “a”).

## Effect-based analysis

### Measured effects using the GeneBLAzer assay (BEQ_bio_)

Figure 1: Example of the highest measured activities in ERα-GeneBLAzer assay found in sample EU001. CRCs for the entire set of samples and all four endpoints can be found in the supporting information (Table Sx).

Figure A4: Example of the highest measured activities in ERα-GeneBLAzer assay found in sample EU001. The concentration response curves (CRCs) for the entire set of samples and all four endpoints can be found in the supporting information (Tables B8-B11).


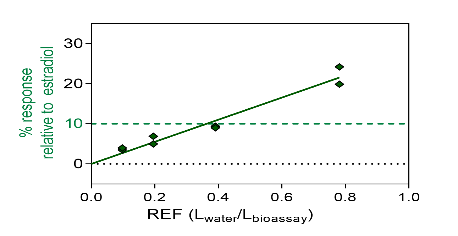

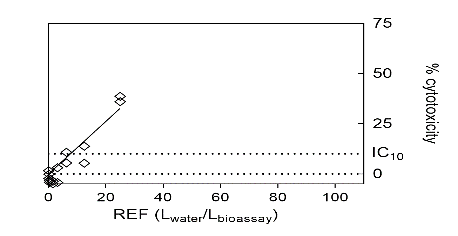

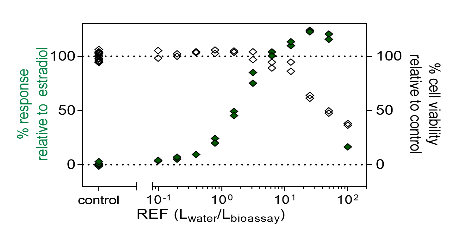


### Measured effects using the GeneBLAzer assay (BEQ_bio_)


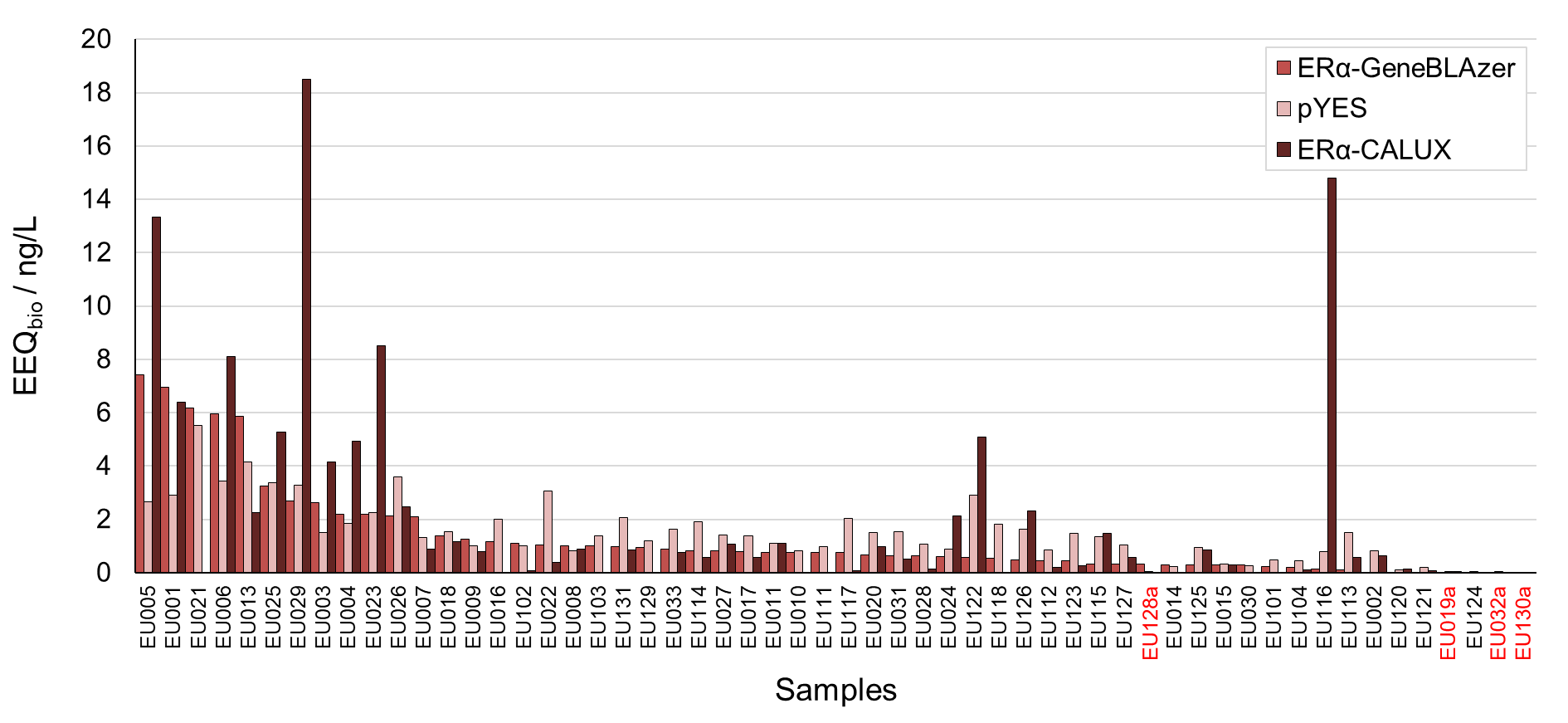


Figure A5: Estrogenic activity detected in three different ERα-bioassays: ERα-GeneBLAzer (of samples without clean-up), p-YES and ERα-CALUX^®^. Samples sorted by ERα-GeneBLAzer results. Samples from WWTPs with advanced treatment are highlighted in red (suffix “a”).

### Predicted effects using chemical analysis (BEQ_chem_)


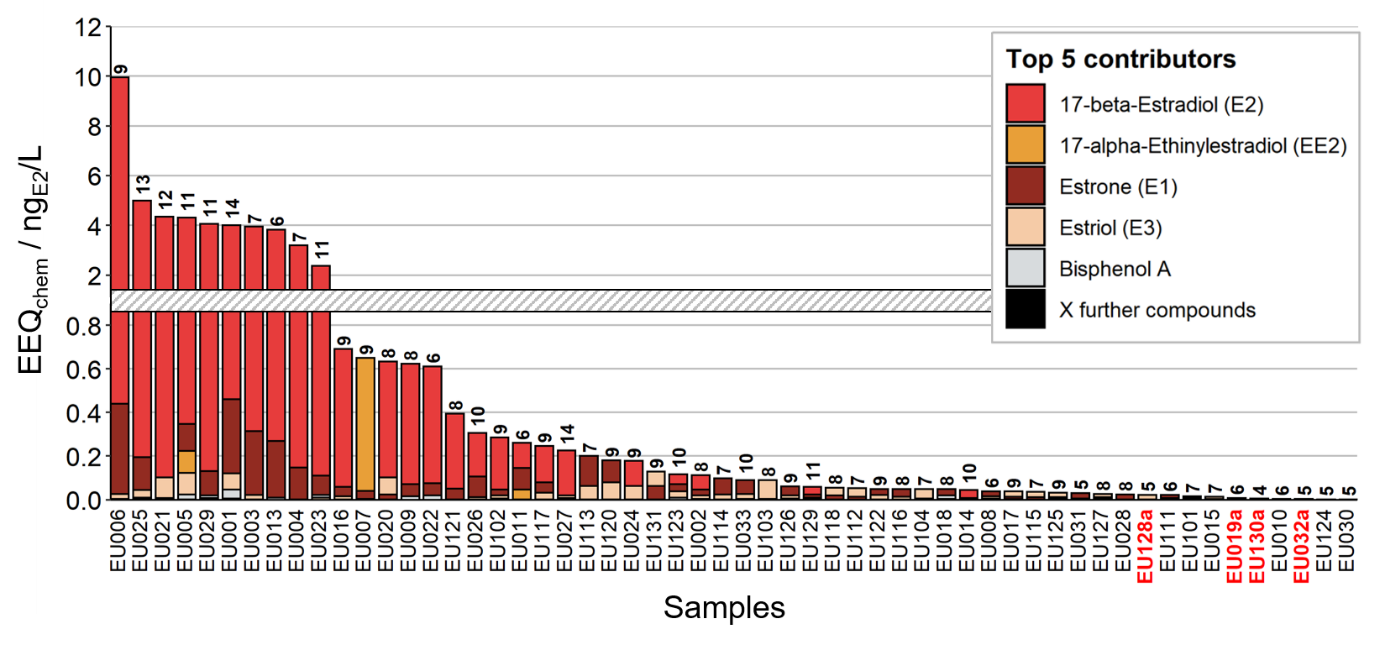


Figure A6: Sum of EEQ_chem,i_ (bars) and number of ERα-active chemicals (above each bar) per sample in the ERα-GeneBLAzer assay. The coloured stacks within the bars represent the individual values for EEQ_chem,i_ of the top 5 contributors by maximum (over all samples), and are sorted by increasing values. Further compounds contributing to EEQ_chem_ are aggregated in the black sack at the bottom of each bar (“X further compounds”, X=15) and listed in the supporting information (Table B6). Samples from WWTPs with advanced treatment are highlighted in red (suffix “a”).


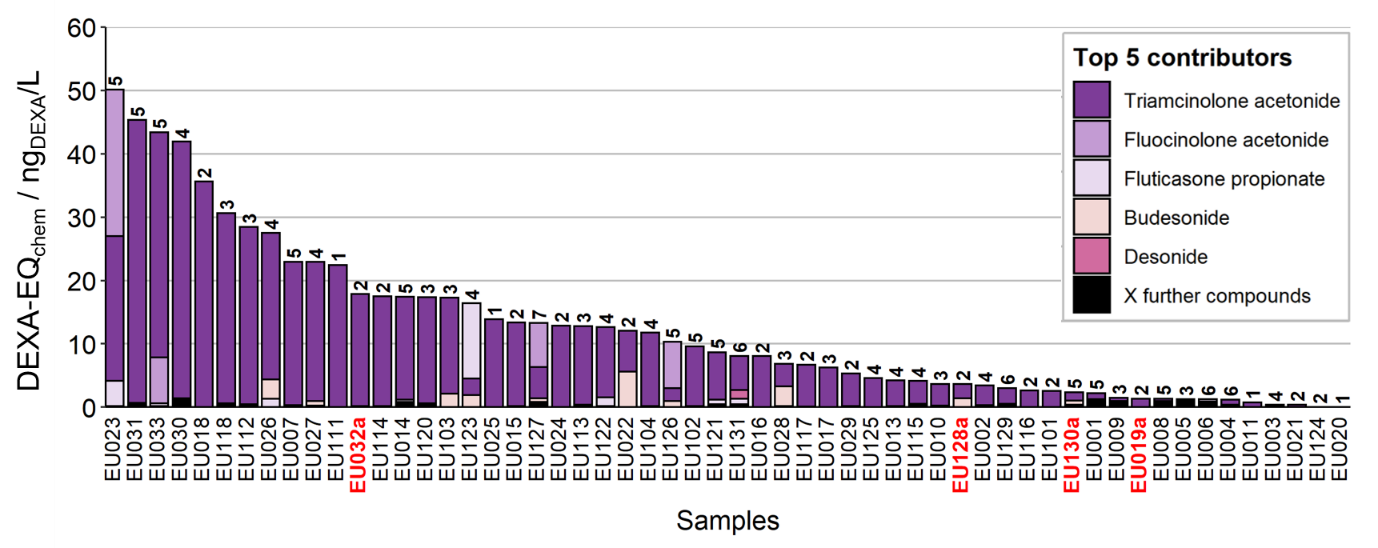


Figure A7: Sum of DEXA-EQ_chem,i_ (bars) and number of GR-active chemicals (above each bar) per sample in the GR-GeneBLAzer assay. The coloured stacks within the bars represent the individual values for DEXA-EQ_chem,i_ of the top 5 contributors by maximum (over all samples), and are sorted by increasing values. Further compounds contributing to DEXA-EQ_chem_ are aggregated in the black stacks at the bottom of each bar (“X further compounds”, X=10) and listed in the supporting information, Table B6. Samples from WWTPs with advanced treatment are highlighted in red (suffix “a”).


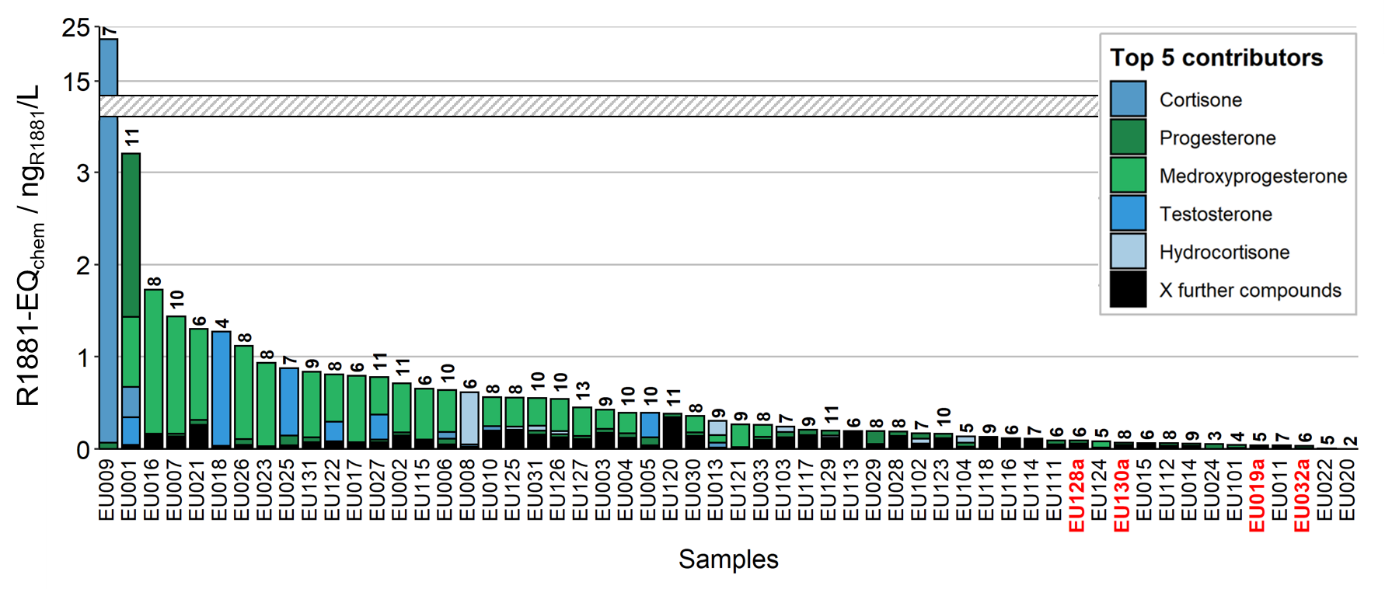


Figure A8: Sum of R1881-EQ_chem,i_ (bars) and number of AR-active chemicals (above each bar) per sample in the AR-GeneBLAzer assay. The coloured stacks within the bars represent the individual values for R1881-EQ_chem,i_ of the top 5 contributors by maximum (over all samples), and are sorted by increasing values. Further compounds contributing to R1881-EQ_chem_ are aggregated in the black stacks at the bottom of each bar (“X further compounds”, X=15) and listed in the supporting information (Table B6). Samples from WWTPs with advanced treatment are highlighted in red (suffix “a”)


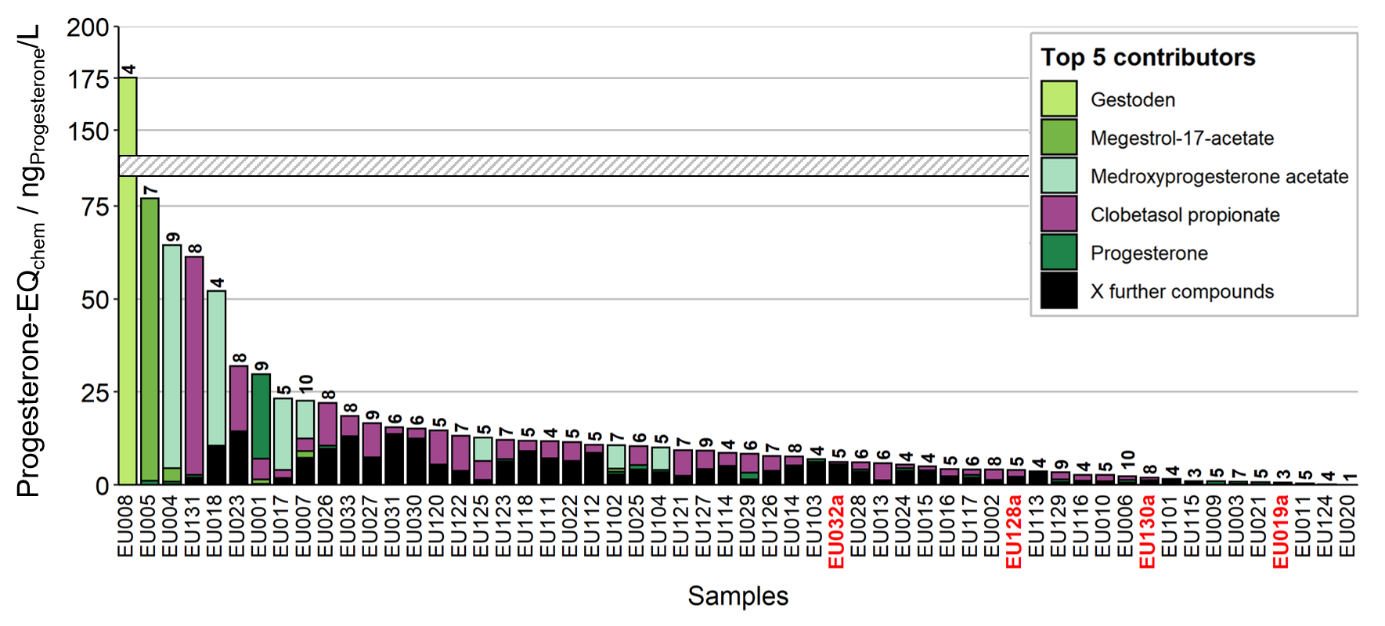


Figure A9: Sum of Progesterone-EQ_chem,i_ (bars) and number of PR-active chemicals (above each bar) per sample in the PR-GeneBLAzer assay. The coloured stacks within the bars represent the individual values for Progesterone-EQ_chem,i_ of the top 5 contributors by maximum (over all samples), and are sorted by increasing values. Further compounds contributing to Progesterone-EQ_chem_ are aggregated in the black stacks at the bottom of each bar (“X further compounds”, X=16) and listed in the supporting information (Table B6). Samples from WWTPs with advanced treatment are highlighted in red (suffix “a”).

## Iceberg Modelling: Linking chemical and effect-based screening


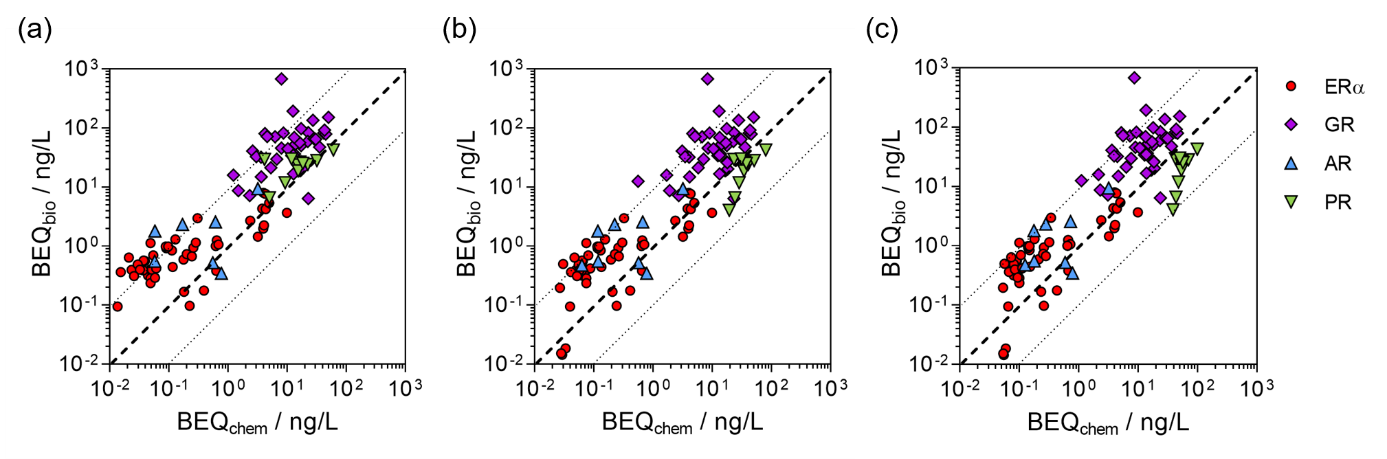


Figure A10: Correlations of BEQ_chem_ and BEQ_bio_ (Iceberg Modelling). (a) Concentrations of compounds detected in at least one WWTP effluent sample, which were not available (NA) were set to zero. (b) NAs were set to half the method detection limit (MDL/2), (c) NAs were set to the method detection limit (MDL).

## Assessment of the endocrine disruptive potential of WWTP effluents

### Derivation of tentative effect-based trigger values

Moving the EBT-EEQ of option 1 (Equation 10) from only three estrogenic compounds in Escher et al. (2018) to 26 compounds in the present study, of which 11 were active in the ERα-GeneBLAer assay and had also available PNEC values, reduced the EBT-EEQ from 0.34 to 0.03 ng_E2_/L. This is the inherent problem if low-potency chemicals are added in the derivation of EBTs, they draw the EBT to lower values rather than higher values, which is one of the reasons why most EBT derivation methods focussed only on high-potency chemicals or introduced a filtering step. Inclusion of the REP ratio for *in-vivo* versus *in-vitro* in option 1 resulted in option 3 (Equation 12) with an EBT-EEQ value of 0.012 ng_E2_/L. In option 2 (Equation 11), fractions were not included, meaning the REP ratio was applied to the core equation, resulting in an EBT-EEQ of 0.20 ng_E2_/L.

The EBTs for GR, AR and PR were previously very uncertain or not defined. In this study, the largely increased database of single chemicals active in the GR-, AR- and PR-GeneBLAzer assays allowed for the first time a derivation of “*in-vivo*/*in-vitro* ratio-corrected” EBTs, based on available PNEC_i_ (and hence available REP*_in‑vivo_*_,i_) for 22, 31 and 21 compounds, respectively. EBTs for GR relied on dexamethasone, dichlorophen and megestrol-17-acetate. For AR, the EBT was based on E1, E2, EE2 and 4-androstene-3,17-dione, and for PR on E2, EE2, megestrol-17-acetate and progesterone. Again, EBTs were calculated according to the abovementioned derivation options and the core equation for GR, AR and PR (Figure A11). The resulting endpoint-specific EBTs according to the different options, ranged roughly within one order of magnitude. The *in-vivo*/*in-vitro* ratio-correction included in options 2 and 3 didn’t tend to affect the EBTs towards systematically lower or higher values. In general, all EBTs are very susceptible to changes in the underlying EQS_i_ (or PNEC_i_) values, especially for high impact compounds. In the specific case of the EBT-EEQ, the EQS of E2 (high REP) changed from 0.4 ng/L in Escher et al. (2018) to now 0.1 ng/L, a difference by a factor of 4.


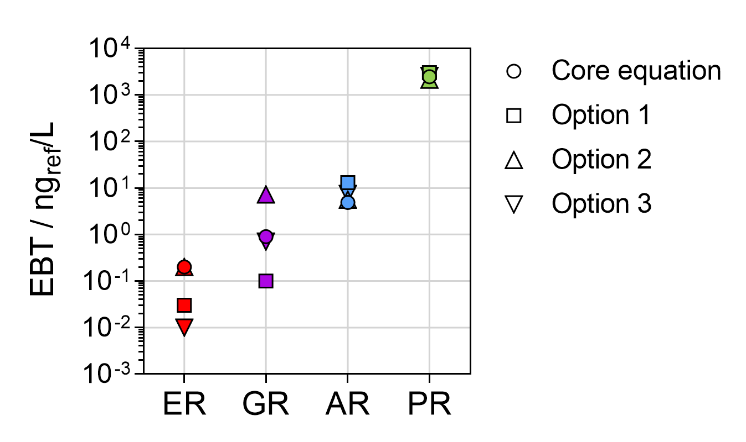


Figure A11: EBT ranges for the GeneBLAzer bioassay according to the core equation and three different EBT derivation options for surface water (i.e. no dilution factor included). ER: EEQ-EBT, GR: DEXA-EQ, AR: R1881-EQ, PR: Progesterone-EQ.

### Assessment of BEQ_bio_ and BEQ_chem_ against tentative thresholds

The application of the GeneBLAzer-specific thresholds for surface water (SW-EBTs) as well as for wastewater (WW-EBT, i.e. incl. dilution factor of 10) to the measured activities (BEQ_bio_) in the investigated WWTP effluent samples resulted in clear differences between the three EBT derivation options (Figure A12a-c). In general, EEQ_bio_ would mostly exceed the WW-EBT (87%, 20% and 98%, for options 1, 2 and 3, respectively). Both for R1881-EQ_bio_ and Progesterone-EQ_bio_ no cases of WW-EBT exceedance were found. For DEXA-EQ_bio_, the situation depended largely on the applied EBT derivation option. In the case of option 2 (Figure A12b), most samples would exceed the SW-EBT (95%) but only a quarter would exceed the WW-EBT (27%), while for options 1 and 3 (Figures A12a and A12c, respectively), all samples would exceed SW-EBT and most would even exceed WW-EBT (100% and 98%, respectively).


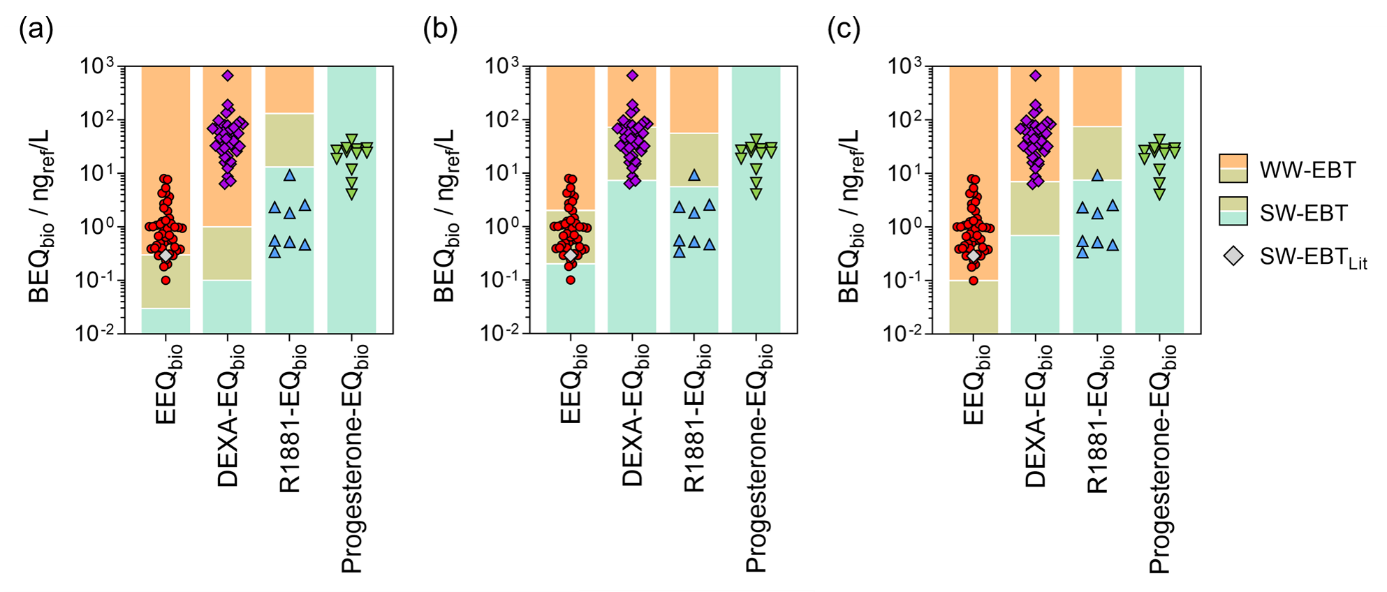


Figure A12: Derived EBT ranges and BEQ_bio_ for the ERα-, GR-, AR- and PR-GeneBLAzer assays, expressed as EEQ_bio_, DEXA-EQ_bio_, R1881-EQ_bio_ and Progesterone-EQ_bio_. EBTs based on (a) Option 1, (b) Option 2 and (c) Option 3. SW-EBT: Threshold for surface water, WW-EBT: Threshold for wastewater, incl. a dilution factor of 10. SW-EBT_Lit_: Mean EBT-EEQ value for the ERα-GeneBLAzer bioassay according to the literature.

Finally, the data basis in the form of measured EQS_i_ and PNEC_i_ values of the substances active in the bioassay must be improved to calculate more robust EBT values. Ideally, the calculation of REP*_in-vivo_*_,i_ according to Equation 4 would not rely on PNEC_i_ values at all, but on measured effect concentrations from targeted *in-vivo* bioassays such as the vitellogenin induction. According to Jobling and Tyler (2003) the induction of the egg yolk protein vitellogenin in male fish is a promising indicator of the presence of estrogens and xenobiotic estrogenic compounds in water. Unfortunately, these data are even less available than reliable EQS_i_ and PNEC_i_ values.

# References

Escher BI, Asmall yi U-AyUS, Behnisch PA, Brack W, Brion F, Brouwer A, et al. Effect-based trigger values for in vitro and in vivo bioassays performed on surface water extracts supporting the environmental quality standards (EQS) of the European Water Framework Directive. Sci Total Environ 2018; 628-629: 748-765. https://doi.org/10.1016/j.scitotenv.2018.01.340.

Jobling S, Tyler CR. Endocrine disruption in wild freshwater fish. Pure and Applied Chemistry 2003; 75: 2219-2234. https://doi.org/10.1351/pac200375112219.

Mosmann T. Rapid colorimetric assay for cellular growth and survival: Application to proliferation and cytotoxicity assays. Journal of Immunological Methods 1983; 65: 55-63. https://doi.org/10.1016/0022-1759(83)90303-4.

Schoenborn A, Schmid P, Bram S, Reifferscheid G, Ohlig M, Buchinger S. Unprecedented sensitivity of the planar yeast estrogen screen by using a spray-on technology. J Chromatogr A 2017; 1530: 185-191. https://doi.org/10.1016/j.chroma.2017.11.009.
